# Supplementary material for: Evolutionary and epidemiological considerations for anthelmintic treatments in ruminant livestock
Source: Int J Parasitol Drugs Drug Resist. 2026 Aug 25;32:100669. doi: 10.1016/j.ijpddr.2026.100669 (PMC13559923; doi:10.1016/j.ijpddr.2026.100669)
Supplement: Supplementary file 1 [file mmc1.pdf]

Supplementary file for Evolutionary epidemiological considerations for the anthelmintic treatments in ruminant livestock: insights from simulation modelling

Authors: Neil Philip Hobbs, John Graham-Brown, Eric Morgan, Hannah Rose Vineer

Contents

S1 Model Equations 2
S1.1 COHORT extension overview: 3
S1.2 AR and AR2 extension overview: 4
S1.3 Model Equations 4
S1.3.1 Model Equations – Resistance Allele Frequency 4
S1.3.2 Model Equations - Eggs 5
S1.3.3 Model Equations – Free-living larvae 8
S1.3.4 Model Equations – Within-host 9
S1.3.5 Model Equations – Symbols and Descriptions 10
S1.3.6 gin function 12
S2 Simulation Set 1 17
S2.1 Overview 17
S2.2 Calibration of Transmission Intensity 17
S2.3 Calculating anthelmintic drug parasite killing efficacy given drug decay:...... 19
S2.4 Conditions varied for sensitivity analysis 19
S2.5 Supplementary Analysis 19
S3 Simulation Set 2 26
S3.1 Overview 26
S3.2 Detailed Parameter Descriptions 26
S3.3 Conditions varied for sensitivity analysis 27
S3.4 Supplementary Analysis 27
S4 GLOWORM Simulation Framework Showcase 32
S4.1 Overview 32

S4.2 Example Simulations 32
S5 Model Validation 37
S5.1 Model Validation Overview 37
S5.2 Validation against Stear Study 37
S5.3 Validation against Sargison Study 39
S5.4 Validation against Waller Study 41
References 43

S1 Model Equations

We further provide the technical extensions of the GLOWORM (Rose et al., 2015; Rose Vineer et al., 2020) framework to include both resistance genetics and multiple livestock cohorts. Previously the GLOWORM framework was methodologically extended to include different patches (META) (Ingle et al., 2025), which allows for the movement of livestock populations between different patches. We now further add the COHORT and AR/AR2 extensions. Note, that META and COHORT can be combined with just the single gene AR extension. Practically, each extension can be used in isolation or together such that the current full GLOWORM framework (Figure S1) is: GLOWORM-META-COHORT-{AR/ AR2}. This flexibility allows for a range of pharmacological interventions to be investigated in an evolutionary epidemiological context (see Section S5 for examples).

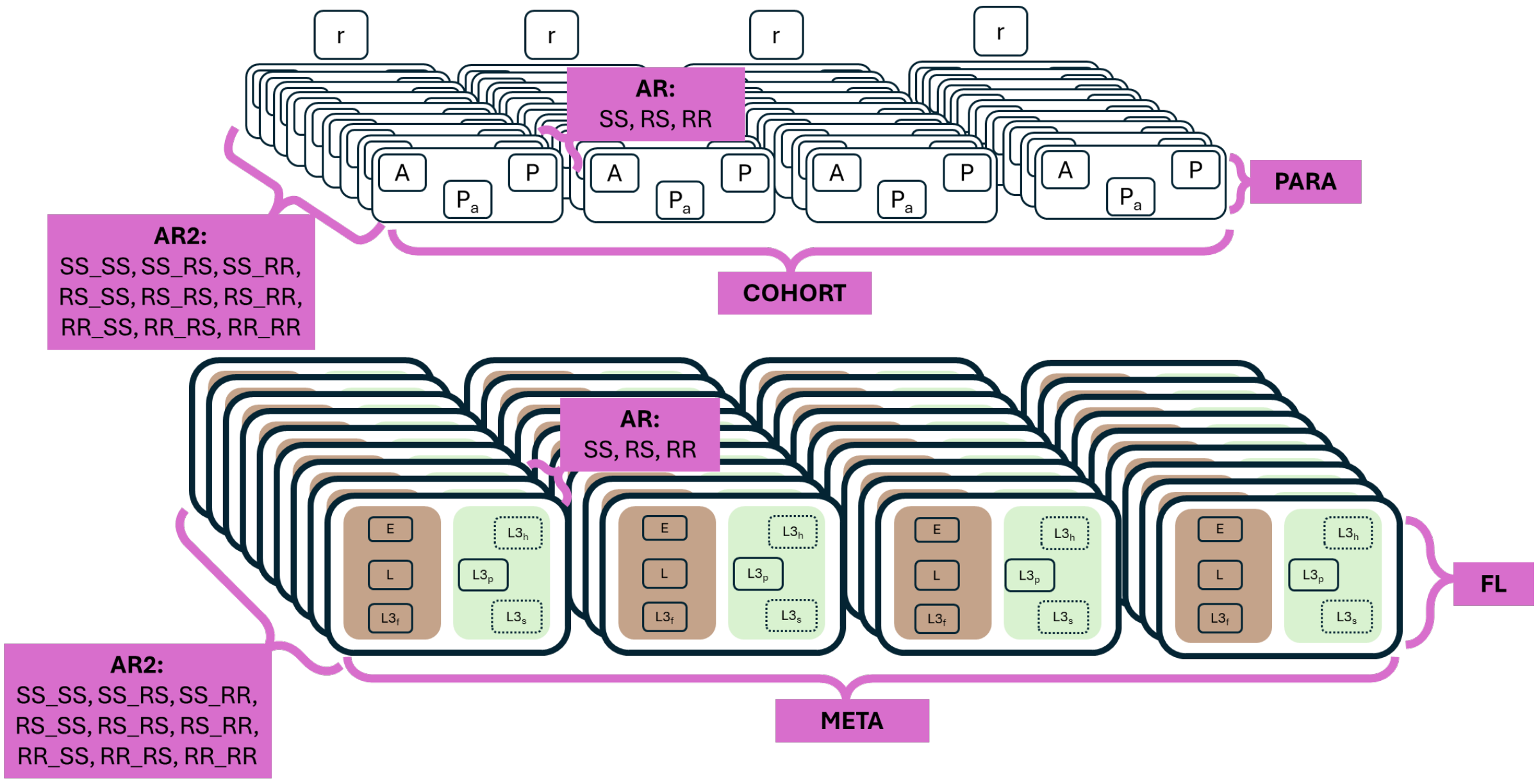

Fig. S1. GLOWORM-META-COHORT-{AR, AR2} Modularisation. This is duplication of Fig. 1 in main manuscript. The GLOWORM model framework consists of a modular format, allowing for the different extensions to be used in unique permutations. The FL (lower half of diagram) (Rose et al., 2015) and PARA (upper half of diagram) (Rose Vineer et al., 2020) are the core parasite transmission components of the model, used across all other model extensions. The META (Ingle et al., 2025) component extends to allow for separate patches for free-living parasites (represented by replication from left to right of the lower half of the diagram). The new COHORT extension allows for separate groups of grazing livestock (represented by replication from left to right in the upper half of the diagram). The AR and AR2 extensions are when the number of different anthelmintic genes being tracked is specified (represented by replication from front to back of the diagram, on the z axis).

S1.1 COHORT extension overview:

The extension to multiple livestock cohorts (COHORT) allows for treatment regimens (and the dynamics this produces) to be specified differently between parents and offspring livestock. Each livestock cohort may have unique properties (age, treatments, movements), and this allows for two key areas to be investigated: explicit treatment *refugia* and parent-offspring treatment dynamics. The livestock cohorts are denoted by the subscript $Cohort$. Each livestock cohort has its own within host dynamics, but different livestock cohorts can exist on the same patch at the same time, be given different treatments at different times, and importantly, the livestock cohorts can be different weights to allow the simultaneous modelling of parents and offspring. Livestock are assigned to one cohort, which they remain in throughout a simulation.

We can extend the model to include separate "cohorts" of livestock. For example, adult livestock and juvenile livestock, who have large discrepancies in weight, acquired immunity, and dry matter intake. We define a "cohort" as a group of livestock which remain constant in their composition throughout any simulation who have the same biological parameters (e.g., weight and dry matter intake), and also receive the same treatments at the same time. Within each simulation, it is possible to give the cohorts

different biological properties, which is especially important when considering the transmission dynamics occurring between parental and offspring livestock. Following from (Ingle et al., 2025), we denote the patches i, and specify the patch any particular cohort is on as j.

S1.2 AR and AR2 extension overview:

The capability to simultaneously and separately model resistance to two different active ingredients is required to evaluate the evolutionary and epidemiological implications of ARM strategies including drug rotations and drug combinations where different active ingredients target the same parasite species. We consider a single gene extension (AR) and a two gene extension (AR2). The genotypes of the parasitic worms are denoted by the superscript $genotype$. In AR $genotype \in \{SS, RS, RR\}$, such that there are 3 permutations, the homozygous susceptible (SS), heterozygote (RS) and homozygous resistant (RR). For the AR2 extension, where there are two genes, $genotype \in \{SS\_SS,\ SS\_RS,\ SS\_RR,\ RS\_SS, RS\_RS,\ RS\_RR,\ RR\_SS,\ RR\_RS,\ RR\_RR\}$, such that there are 9 possible genotype permutations when considering a two-gene system. The first two letters (before the underscore) are for the first gene (which would be resistance to drug A), and the two letters (after the underscore) correspond to the second gene (which would be resistance to drug B). As is common in the resistance modelling literature we use S for susceptible, and R for resistant. The assumption of monogenic resistance is widespread throughout the resistance modelling literature regardless of biological system (e.g., (Helps et al., 2020; Levick et al., 2017; Madgwick and Kanitz, 2022; Reichert et al., 2023; REX Consortium, 2013)).

S1.3 Model Equations

S1.3.1 Model Equations – Resistance Allele Frequency

The distribution of the resistance genotypes of the eggs laid will depend on the resistance allele frequency of the adult worm population, which resides in the various livestock cohorts. Because each cohort can have unique patch movements and drug treatments the frequency the resistance allele frequency may differ between cohorts, and therefore must be calculated separately for each cohort separately. We can then calculate the resistance allele frequency (q) for each gene in the adult worm population, and this is conducted separately for each livestock cohort. Such that ${q_1}_{Cohort}$ refers to the resistance allele frequency of gene 1 in the adult worm population in the livestock cohort $Cohort$, and ${q_2}_{Cohort}$ refers to the corresponding resistance allele frequency of gene 2. We must track the resistance allele frequencies in the adult worm population in the cohorts separately because different resistance allele frequencies may arise given different management practices between the livestock cohorts. The resistance allele frequencies in the livestock cohorts are subsequently used to calculate the distribution of the genotypes of the eggs laid by the adult worm population.

$$q_{1\,Cohort(t)} = \frac{\left(A^{RR\_RR}_{Cohort(t)} + A^{RR\_RS}_{Cohort(t)} + A^{RR\_SS}_{Cohort(t)} + \left(0.5 * \left(A^{RS\_RR}_{Cohort(t)} + A^{RS\_RS}_{Cohort(t)} + A^{RS\_SS}_{Cohort(t)}\right)\right)\right)}{\left(\sum_{genotype=1}^{n.genotypes} A^{genotype}_{Cohort(t)}\right)}$$

Equation S1a

This is similarly calculated for the second gene:

$$q_{2\,Cohort(t)} = \frac{\left(A^{RR\_RR}_{Cohort(t)} + A^{RS\_RR}_{Cohort(t)} + A^{SS\_RR}_{Cohort(t)} + \left(0.5 * \left(A^{RR\_RS}_{Cohort(t)} + A^{RS\_RS}_{Cohort(t)} + A^{SS\_RS}_{Cohort(t)}\right)\right)\right)}{\left(\sum_{genotype=1}^{n.genotypes} A^{genotype}_{Cohort(t)}\right)}$$

Equation S1b

Note, the model can readily be adapted to track only a single gene:

$$q_{1\,Cohort(t)} = \frac{\left(A^{RR}_{Cohort(t)} + \left(0.5 * A^{RS}_{Cohort(t)}\right)\right)}{\left(\sum_{genotype=1}^{n.genotypes} A^{genotype}_{Cohort(t)}\right)}$$

Equation S1c

S1.3.2 Model Equations - Eggs

We next update the free-living worm equations for the eggs (Equations 2a to 2i) and free-living larval stages (Equation 3 to Equation 5). These equations can be adapted to a single gene format by removing all the q_2 related terms.

$$\frac{dE_i^{RR\_RR}}{dt} = -(2\delta_1 + \mu_1)E_i^{RR\_RR} + \sum_{Cohort=1}^{n.cohorts}\left(\left(\sum_{genotype=1}^{n.genotypes} \lambda A^{genotype}_{j\ Cohort} H_{ij} C_e\right)\left({q_{1\,Cohort}}^2 {q_{2\,Cohort}}^2\right)\right)$$

Equation S2a(i)

This equation tracks the number of eggs on patch i, which have the genotype RR_RR. $\left(\sum_{genotype=1}^{n.genotypes} \lambda A^{genotype}_{j\ Cohort} H_{ij} C_e\right)$ calculates the total number of eggs laid by a cohort on

a patch, which is then converted to the number of the tracked genotype (in this case for the RR_RR genotype using ${q_1}_{Cohort}^2 {q_2}_{Cohort}^2$). This is then wrapped in the $\sum_{Cohort}^{n.cohorts}$ such that the total number of new eggs are counted across all cohorts.

These equations can be adapted to a single gene format by removing all the q_2 related terms, for example:

$$\frac{dE_i^{RR}}{dt} = -(2\delta_1 + \mu_1)E_i^{RR} + \sum_{Cohort=1}^{n.cohorts}\left(\left(\sum_{genotype=1}^{n.genotypes} \lambda A_{j\,Cohort}^{genotype} H_{ij} C_e\right)\left({q_1}_{Cohort}^2\right)\right)$$

Equation S2a(ii)

The following egg equations are similarly as above, differing in which genotype being tracked.

$$\frac{dE_i^{RR\_RS}}{dt} = -(2\delta_1 + \mu_1)E_i^{RR\_RS} + \sum_{Cohort=1}^{n.cohorts}\left(\left(\sum_{genotype=1}^{n.genotypes} \lambda A_{j\,Cohort}^{genotype} H_{ij} C_e\right)\left({q_1}_{Cohort}^2 (2 * \left(1 - {q_2}_{Cohort}\right){q_2}_{Cohort})\right)\right)$$

Equation S2b

$$\frac{dE_i^{RR\_SS}}{dt} = -(2\delta_1 + \mu_1)E_i^{RR\_SS} + \sum_{Cohort=1}^{n.cohorts}\left(\left(\sum_{genotype=1}^{n.genotypes} \lambda A_{j\,Cohort}^{genotype} H_{ij} C_e\right)\left({q_1}_{Cohort}^2 \left(\left(1 - {q_2}_{Cohort}\right)^2\right)\right)\right)$$

Equation S2c

$$\frac{dE_i^{RS\_RR}}{dt} = -(2\delta_1 + \mu_1)E_i^{RS\_RR} + \sum_{Cohort=1}^{n.cohorts}\left(\left(\sum_{genotype=1}^{n.genotypes}\lambda A_{j\,Cohort}^{genotype} H_{ij} C_e\right)\left(\left(2 * \left(1 - q_{1_{Cohort}}\right) q_{1_{Cohort}}\right) q_{2_{Cohort}}{}^2\right)\right)$$

Equation S2d

$$\frac{dE_i^{RS\_RS}}{dt} = -(2\delta_1 + \mu_1)E_i^{RS\_RS} + \sum_{Cohort=1}^{n.cohorts}\left(\left(\sum_{genotype=1}^{n.genotypes}\lambda A_{j\,Cohort}^{genotype} H_{ij} C_e\right)\left(\left(2 * \left(1 - q_{1_{Cohort}}\right) q_{1_{Cohort}}\right)\left(2 * \left(1 - q_{2_{Cohort}}\right) q_{2_{Cohort}}\right)\right)\right)$$

Equation S2e

$$\frac{dE_i^{RS\_SS}}{dt} = -(2\delta_1 + \mu_1)E_i^{RS\_SS} + \sum_{Cohort=1}^{n.cohorts}\left(\left(\sum_{genotype=1}^{n.genotypes}\lambda A_{j\,Cohort}^{genotype} H_{ij} C_e\right)\left(\left(2 * \left(1 - q_{1_{Cohort}}\right) q_{1_{Cohort}}\right)\left(\left(1 - q_{2_{Cohort}}\right)^2\right)\right)\right)$$

Equation S2f

$$\frac{dE_i^{SS\_RR}}{dt} = -(2\delta_1 + \mu_1)E_i^{SS\_RR} + \sum_{Cohort=1}^{n.cohorts}\left(\left(\sum_{genotype=1}^{n.genotypes}\lambda A_{j\,Cohort}^{genotype} H_{ij} C_e\right)\left(\left(1 - q_{1_{Cohort}}\right)^2\right) q_{2_{Cohort}}{}^2\right)$$

Equation S2g

$$\frac{dE_i^{SS\_RS}}{dt} = -(2\delta_1 + \mu_1)E_i^{SS\_RS} + \sum_{Cohort=1}^{n.cohorts}\left(\left(\sum_{genotype=1}^{n.genotypes} \lambda A_{j\,Cohort}^{genotype} H_{ij} C_e\right)\left(\left(1 - q_{1_{Cohort}}\right)^2\right)\left(2 * \left(1 - q_{2_{Cohort}}\right) q_{2_{Cohort}}\right)\right)$$

Equation S2h

$$\frac{dE_i^{SS\_SS}}{dt} = -(2\delta_1 + \mu_1)E_i^{SS\_SS} + \sum_{Cohort=1}^{n.cohorts}\left(\left(\sum_{genotype=1}^{n.genotypes} \lambda A_{j\,Cohort}^{genotype} H_{ij} C_e\right)\left(\left(1 - q_{1_{Cohort}}\right)^2\right)\left(1 - q_{2_{Cohort}}\right)^2\right)$$

Equation S2i

S1.3.3 Model Equations – Free-living larvae

The larval (L1-L2) equation is also updated to account for all genotypes, but can be written in a generalisable format, and is therefore the same equation as Equation 2 in (Rose et al., 2015) except for the extension to include the resistance genotype as a superscript, and the patch location denoted in the subscript.

$$\frac{dL_i^{genotype}}{dt} = -(2\delta_1 + \mu_2)L_i^{genotype} + 2\delta_1 E_i^{genotype}$$

Equation S3

For the L3 larvae in faeces this can also be written in a generalisable format, and is therefore the same equation as Equation 3 in (Rose et al., 2015) except for the

extension to include the resistance genotype as denoted in the superscript, and the patch location denoted in the subscript.

$$\frac{dL3f_i^{genotype}}{dt} = -(m_1 + \mu_3)L3f_i^{genotype} + 2\delta_1 L_i^{genotype}$$

Equation S4

The number of larvae on pasture equation is therefore updated to account for the ability for different cohorts to feed on the pasture. It is key to note that we allow the dry matter intake to be different for each cohort (as specified by β_{Cohort}). Here, as the equations for each genotype would be the same we write this as a generalisable form:

$$\frac{dL3p_i^{genotype}}{dt} = -\mu_4 L3p_i^{genotype}(1 - m_2) - \mu_5 L3p_i^{genotype} m_2 + m_1 L3p_i^{genotype} - \sum_{Cohort=1}^{n.Cohorts} L3p_i^{genotype} \beta_{Cohort} H_{ij}$$

Equation S5

S1.3.4 Model Equations – Within-host

We next update the parasitic life-stages of the equations. For the parasitic life-stages (P, Pa, A), we must track the genotype of the worm (superscript Genotype), the cohort the worm resides in (subscript Cohort) and the patch the Cohort in on (subscript j).

$$\frac{dP_{j\,Cohort}^{genotype}}{dt} = -(\delta_2 + \mu_6)P_{j\,Cohort}^{genotype} + L3p_{ij}^{genotype} m_2 \beta_{Cohort}$$

Equation S6

$$\frac{dPa_{j\,Cohort}^{genotype}}{dt} = -(h_2 + \mu_7)Pa_{j\,Cohort}^{genotype} + \delta_2 P_{j\,Cohort}^{genotype} h_1$$

Equation S7

$$\frac{dA_{j\,Cohort}^{genotype}}{dt} = -\mu_8 A_{j\,Cohort}^{genotype} + \delta_2 P_{j\,Cohort}^{genotype}(1 - h_1) + h_2 Pa_{j\,Cohort}^{genotype}$$

Equation S8

$A_{j\ Cohort}^{genotype}$ is therefore the number of adult worms of genotype $genotype$ infecting the livestock cohort $cohort$ who reside on patch j. $P_{j\ Cohort}^{genotype}$ and $Pa_{j\ Cohort}^{genotype}$ describe the same except for the Parasitic and Arrested life stages as previously detailed (Rose Vineer et al., 2020). It must be emphasised that the $genotype$ superscript refer to the genotype of the worms infecting the cohort and not the genotype of the livestock cohort itself.

As each cohort can be given unique patch movements, treatment regimens or differences in dry matter intake (due to weight differences), immunity is calculated separately for each cohort.

$$\frac{dr_{j\ Cohort}}{dt} = -r_{j\ Cohort}\sigma + \rho(1 - r_{j\ Cohort}) \sum_{genotype=1}^{n.genotypes} L3p_i^{genotype} \beta_{Cohort} m_2$$

Equation S9

Other models have called this livestock immunity to parasites as "resistance"; but for clarity we use the term resistance only when describe a genetic process.

S1.3.5 Model Equations – Symbols and Descriptions

**Table S1.1 Descriptions of the Symbols used in the Equations**	
Symbol	Description
State Variables: values are calculated within the model	
$q_{1\,Cohort(t)}$	The resistance allele frequency for resistance to the first anthelmintic drug (subscript 1) in adult worms in a specific livestock cohort (subscript cohort) at the specified timepoint (t).
$q_{2\,Cohort(t)}$	The resistance allele frequency for resistance to the second anthelmintic drug (subscript 2) in adult worms in a specific livestock cohort (subscript cohort) at the specified timepoint (t).
$A_{Cohort(t)}^{RR\_RR}$	Adult nematodes in a livestock cohort (subscript), with genotype RR_RR (superscript) at the specified timepoint (t).
$A_{Cohort(t)}^{RR\_RS}$	Adult nematodes in a livestock cohort (subscript), with genotype RR_RS (superscript) at the specified timepoint (t).
$A_{Cohort(t)}^{RR\_SS}$	Adult nematodes in a livestock cohort (subscript), with genotype RR_SS (superscript) at the specified timepoint (t).
$A_{Cohort(t)}^{RS\_RR}$	Adult nematodes in a livestock cohort (subscript), with genotype RS_RR (superscript) at the specified timepoint (t).
$A_{Cohort(t)}^{RS\_RS}$	Adult nematodes in a livestock cohort (subscript), with genotype RS_RS (superscript) at the specified timepoint (t).
$A_{Cohort(t)}^{RS\_SS}$	Adult nematodes in a livestock cohort (subscript), with genotype RS_SS (superscript) at the specified timepoint (t).
$A_{Cohort(t)}^{SS\_RR}$	Adult nematodes in a livestock cohort (subscript), with genotype SS_RR (superscript) at the specified timepoint (t).
$A_{Cohort(t)}^{SS\_RS}$	Adult nematodes in a livestock cohort (subscript), with genotype SS_RS (superscript) at the specified timepoint (t).
$A_{Cohort(t)}^{SS\_SS}$	Adult nematodes in a livestock cohort (subscript), with genotype SS_SS (superscript) at the specified timepoint (t).
$A_{Cohort(t)}^{RR\_RR}$	Adult nematodes in a livestock cohort (subscript), with genotype RR_RR (superscript) at the specified timepoint (t).
$A_{Cohort}^{genotype}$	Generalisable form of the descriptions above (e.g., $A_{Cohort(t)}^{RR\_SS}$ or $A_{Cohort(t)}^{RS\_SS}$) of Adult nematodes in a livestock cohort (subscript), with the genotype which can be $genotype \in \{SS\_SS,\ SS\_RS,\ SS\_RR,\ RS\_SS, RS\_RS,\ RS\_RR,\ RR\_SS,\ RR\_RS,\ RR\_RR\}$ (superscript).

$E_i^{RR\_RR}$	Eggs in faeces on patch i (subscript), with the genotype RR_RR (superscript)
$E_i^{RR\_RS}$	Eggs in faeces on patch i (subscript), with the genotype RR_RS (superscript)
$E_i^{RR\_SS}$	Eggs in faeces on patch i (subscript), with the genotype RR_SS (superscript)
$E_i^{RS\_RR}$	Eggs in faeces on patch i (subscript), with the genotype RS_RR (superscript)
$E_i^{RS\_RS}$	Eggs in faeces on patch i (subscript), with the genotype RS_RS (superscript)
$E_i^{RS\_SS}$	Eggs in faeces on patch i (subscript), with the genotype RS_SS (superscript)
$E_i^{SS\_RR}$	Eggs in faeces on patch i (subscript), with the genotype SS_RR (superscript)
$E_i^{SS\_RS}$	Eggs in faeces on patch i (subscript), with the genotype SS_RS (superscript)
$E_i^{SS\_SS}$	Eggs in faeces on patch i (subscript), with the genotype SS_SS (superscript)
$E_i^{genotype}$	Generalisable form of the descriptions above (e.g., $E_i^{RR\_RR}$ or $E_i^{RS\_RS}$) of Eggs in faeces on patch i (subscript), with the genotype which can be $genotype \in \{SS\_SS, SS\_RS, SS\_RR, RS\_SS, RS\_RS, RS\_RR, RR\_SS, RR\_RS, RR\_RR\}$ (superscript).
$L_i^{genotype}$	L1 and L2 in faeces in patch i (subscript) with the genotype which can be $genotype \in \{SS\_SS, SS\_RS, SS\_RR, RS\_SS, RS\_RS, RS\_RR, RR\_SS, RR\_RS, RR\_RR\}$ (superscript).
$L3f_i^{genotype}$	L3 in faeces (denoted by f) in patch i (subscript) with the genotype which can be $genotype \in \{SS\_SS, SS\_RS, SS\_RR, RS\_SS, RS\_RS, RS\_RR, RR\_SS, RR\_RS, RR\_RR\}$ (superscript).
$L3p_i^{genotype}$	L3 on pasture (denoted by p) in faeces in patch i (subscript) with the genotype which can be $genotype \in \{SS\_SS, SS\_RS, SS\_RR, RS\_SS, RS\_RS, RS\_RR, RR\_SS, RR\_RS, RR\_RR\}$ (superscript).
$P_{j\ Cohort}^{genotype}$	Pre-adult parasitic stages (P) in livestock who are on patch j, where the Pre-adult parasitic stages are in a specific cohort (subscript) and have the $genotype \in \{SS\_SS, SS\_RS, SS\_RR, RS\_SS, RS\_RS, RS\_RR, RR\_SS, RR\_RS, RR\_RR\}$ (superscript).
$Pa_{j\ Cohort}^{genotype}$	Hypobiotic larvae (Pa) in livestock who are on patch j, where the Hypobiotic larvae are in a specific cohort (subscript) and have the $genotype \in \{SS\_SS, SS\_RS, SS\_RR, RS\_SS, RS\_RS, RS\_RR, RR\_SS, RR\_RS, RR\_RR\}$ (superscript).
$A_{j\ Cohort}^{genotype}$	Adult nematodes (A) in livestock who are on patch j, where the adult nematodes are in a specific cohort (subscript) and have the $genotype \in \{SS\_SS, SS\_RS, SS\_RR, RS\_SS, RS\_RS, RS\_RR, RR\_SS, RR\_RS, RR\_RR\}$ (superscript).
$r_{j\ Cohort}$	Host (livestock) immunity, for a specific cohort (subscript) on a specific patch (subscript).
Parameters, where value estimates are detailed in (Ingle et al., 2025; Rose et al., 2015; Rose Vineer et al., 2020). Full details of the parameter values for Haemonchus contortus and *Teladorsagia circumcincta* are given in the model code in the "gin" function (duplicated in S1.3.6).	
δ_1	*E* to L3f development rate
μ_1	E mortality rate
λ	Per A fecundity rate
H_{ij}	Host stocking density
C_e	Egg correction factor
μ_2	L mortality rate
m_1	L3f horizontal migration rate
μ_3	L3f mortality rate
μ_4	L3s mortality rate
μ_5	L3f mortality rate
m_2	L3p vertical migration rate
μ_5	L3h mortality rate
β_{Cohort}	Host (Cohort) herbage ingestion rate
δ_2	P to A development rate
μ_6	P mortality rate
μ_7	Pa mortality rate
h_2	Emergence rate
h_1	Hypobiosis rate
μ_8	A mortality rate
σ	Immune decay rate
ρ	Immune response per L3 ingested

S1.3.6 gin function

Printed out below is the gin function (written in R) which details the parameter values used for the respective parasite species, and therefore shows exactly how those values are calculated.

```
#' Get GI nematode life history parameters for the GLOWORM models
#'
#' This function defines the life history parameters needed as input for t
he gloworm models, dependent on nematode species and environmental conditi
ons.
#' This function can be used alone to extract the parameters, but it is al
so called by the GLOWORM model functions, and therefore you do not have to
use this function prior to running the models.
#'
#' @param nematode 1 = Haemonchus contortus, 2 = Teladorsagia circumcinta,
3 = Ostertagia ostertagi, 4 = Cooperia oncophora
#' @param temp daily time series of mean temperature. See ?gloworm::eobspo
int for a possible method to obtain these data
#' @param precip daily time series of total rainfall. See ?gloworm::eobspo
int for a possible method to obtain these data
#' @param photoperiod daily time series of daylight hours. See ?geosphere:
:daylength for a possible method to obtain these data
#' @return Data frame with species-specific life history parameters, to be
used as input for the GLOWORM model functions
#' @examples
#' gin(nematode = 2, temp = rep(10, 10), precip = rep(5, 10), photoperiod
= rep(12, 10))
#' @importFrom imputeTS na.ma
#' @importFrom forecast ma
#' @export

gin = function(nematode, temp, precip, photoperiod) {


  if(nematode==1) {


    PET = 0.55 * ((photoperiod/12)^2) * ((4.95 * exp(0.062 * temp))/100) *
25.4
    PET_lag = NA
    PETm_lag = NA
    Precip_lag = NA
    Precipm_lag = NA
    for (z in 1:length(PET)) {
      minday = z
      maxday = ifelse((z + 4) > length(PET), length(PET),
                      z + 4)
      PET_lag[z] = sum(x = PET[minday:maxday])
      Precip_lag[z] = sum(x = precip[minday:maxday])
      maxday = z
      minday = ifelse((z - 4) < 1, 1, z - 4)
```

```
      PETm_lag[z] = sum(x = PET[minday:maxday])
      Precipm_lag[z] = sum(x = precip[minday:maxday])
      P.E.1 = Precip_lag/PET_lag
      P.E.2 = Precipm_lag/PETm_lag
    }

    dev.1 = pmin(1, pmax(0, -0.09746 + 0.01063*temp))
    mu.1 = pmin(1, exp(-1.47135 -0.11444*temp + 0.00327*(temp^2)))
    mu.2 = pmin(1, exp(-1.82300 -0.14180*temp + 0.00405*(temp^2)))
    mu.3 = pmin(1, exp(-2.63080 - 0.14407*temp + 0.00463*(temp^2)))
    mu.4 = pmin(1, exp(-3.68423  - 0.2535 *temp + 0.00740 *(temp^2)))
    mu.5 = mu.3
    h.mig = ifelse(precip>=2, 0.25, ifelse(P.E.2>=1, 0.051, 0))
    v.mig = (pmax(0, exp(-5.48240 + 0.45392*temp -0.01252*(temp^2))))

    egg.correction = ifelse(P.E.1 < 1, 0.1, 1)

    dev.2 = -log(0.5)/17
    max.mu6 = -log(0.24)/17
    min.mu6 = -log(0.45)/17
    mu7 = 0.002
    min.mu8 = 0.021-(1.96*0.002)
    max.mu8 = 0.021
    min.h = 0
    max.h = 0.50
    min.lambda = log(1295/2)
    max.lambda = log((1295.9+(1.96*280.4))/2)
    rho.response = 9e-4
    sigma.decay = -log(0.1)/84
    pAf = 0.5

    devsuccess = devsuccess = (dev.1*h.mig)/((mu.1+mu.2+dev.1)*(mu.3+h.mig
))
    hypobiosis = max.h - ((max.h-min.h)/(max(devsuccess, na.rm = TRUE)))*(
devsuccess) # (max(devsuccess, na.rm = TRUE)-min(devsuccess, na.rm = TRUE)
))*(devsuccess-min(devsuccess, na.rm = TRUE)) #max.h - ((max.h-min.h)/(max
(photoperiod)-min(photoperiod)))*(photoperiod-min(photoperiod))
    resume = (1/(max(devsuccess, na.rm = TRUE)-min(devsuccess, na.rm = TRU
E)))*(devsuccess-min(devsuccess, na.rm = TRUE))

    print('Haemonchus parameters sourced')

    (sapply(ls(),function(x)get(x),simplify=F,USE.NAMES=T))

  } else {

    if(nematode==2) {
      # T. circumcincta

      PET = 0.55 * ((photoperiod/12)^2) * ((4.95 * exp(0.062 * temp))/100)
* 25.4
      PET_lag = NA
      PETm_lag = NA
      Precip_lag = NA
```

```
  Precipm_lag = NA
  for (z in 1:length(PET)) {
    minday = z
    maxday = ifelse((z + 7) > length(PET), length(PET),
                    z + 7)
    PET_lag[z] = sum(x = PET[minday:maxday])
    Precip_lag[z] = sum(x = precip[minday:maxday])
    maxday = z
    minday = ifelse((z - 7) < 1, 1, z - 7)
    PETm_lag[z] = sum(x = PET[minday:maxday])
    Precipm_lag[z] = sum(x = precip[minday:maxday])
    P.E.1 = Precip_lag/PET_lag
    P.E.2 = Precipm_lag/PETm_lag
  }
  dev.1 = pmin(1, pmax(0, -0.02085 + 0.00467 * temp))
  mu.1 = pmin(1, exp(-1.62026 - 0.17771 * temp + 0.00629 * (temp^2)))
  mu.2 = mu.1
  mu.4 = pmin(1, exp(-4.58817 - 0.13996 * temp + 0.00461 * (temp^2)))
  mu.3 = pmin(1, 10 * mu.4)
  mu.5 = mu.3
  h.mig = ifelse(precip >= 2, 0.21, ifelse(P.E.2 >= 1, 0.025, 0))
  v.mig = (pmax(0, exp(-5.4824 + 0.45392 * temp - 0.01252 * (temp^2)))
)

  egg.correction = ifelse(P.E.1 < 1, 0.1, 1)

  dev.2 = -log(0.5)/17
  max.mu6 = -log(0.0107)/17
  min.mu6 = -log(0.3399)/17
  mu7 = 0.002
  min.mu8 =  -log(0.97)
  max.mu8 = -log(0.89)
  min.h = 0
  max.h = 0.35
  min.lambda = log(0.025*500)
  max.lambda = log(0.375*500)
  rho.response = 1e-4
  sigma.decay = -log(0.5)/60
  pAf = 0.5 # Can probably remove this?

  devsuccess = (dev.1*h.mig)/((mu.1+mu.2+dev.1)*(mu.3+h.mig))
  hypobiosis = max.h - ((max.h-min.h)/(max(devsuccess, na.rm = TRUE)))
*(devsuccess) # (max(devsuccess, na.rm = TRUE)-min(devsuccess, na.rm = TRU
E)))*(devsuccess-min(devsuccess, na.rm = TRUE)) #max.h - ((max.h-min.h)/(m
ax(photoperiod)-min(photoperiod)))*(photoperiod-min(photoperiod))
  resume = (1/(max(devsuccess, na.rm = TRUE)-min(devsuccess, na.rm = T
RUE)))*(devsuccess-min(devsuccess, na.rm = TRUE))

  print('Teladorsagia parameters sourced')

  (sapply(ls(),function(x)get(x),simplify=F,USE.NAMES=T))

} else {
```

```
      if(nematode==3) {
        # O ostertagia

        dev.1 = pmin(1, pmax(0, -0.07258 + 0.00976*temp))
        mu.1 = pmin(1, exp(-4.38278 -0.10640*temp + 0.00540*(temp^2)))
        mu.2 = pmin(1, exp(-4.38278 -0.10640*temp + 0.00540*(temp^2)))
        mu.4 = pmin(1, exp(-5.743  - 0.1755 *temp + 0.0068 *(temp^2) -0.00003*(temp^3))) # pmin(1, exp(-6.388  - 0.2681 *temp + 0.01633 *(temp^2) -0.00016*(temp^3)))
        mu.3 = pmin(1, exp(-4.864  - 0.206 *temp + 0.0048 *(temp^2) +0.00008*(temp^3))) # pmin(1, mu.4*10)
        mu.5 = mu.3
        h.mig = ifelse(precip>=5, pmax(0, pmin(1, exp(-3.29608 + 0.04594*precip))),0)
        v.mig = (pmax(0, exp(-5.48240 + 0.45392*temp - 0.01252*(temp^2))*0.268))

        egg.correction = rep(1, length(temp))

        dev.2 = 0.04077336
        max.mu6 = -log(0.23)/17 # PPP of 17 days, low establishment rate of 0.05% in Verschave
        min.mu6 = 0.054 # -log(0.27)/17 # PPP 17 days, mean establishment rate of 0.27
        mu7 = 0.002
        min.mu8 = 0.028 # mean adult mortality rate verschave
        max.mu8 = 0.08 # high adult mortality rate verschave
        min.h = 0.02
        max.h = 0.3 # min max range in Verschave
        min.lambda = 4.58
        max.lambda = 4.96
        rho.response = 5.981e-5
        sigma.decay = 0.002
        pAf = 0.5

        devsuccess = as.numeric(na.ma(ma(dev.1, order = 7,centre = FALSE), k = 7))
        hypobiosis = max.h - ((max.h-min.h)/(max(devsuccess, na.rm = TRUE)))*(devsuccess) # (max(devsuccess, na.rm = TRUE)-min(devsuccess, na.rm = TRUE)))*(devsuccess-min(devsuccess, na.rm = TRUE)) #max.h - ((max.h-min.h)/(max(photoperiod)-min(photoperiod)))*(photoperiod-min(photoperiod))
        resume = (1/(max(devsuccess, na.rm = TRUE)-min(devsuccess, na.rm = TRUE)))*(devsuccess-min(devsuccess, na.rm = TRUE))

        print('Ostertagia parameters sourced')

        (sapply(ls(),function(x)get(x),simplify=F,USE.NAMES=T))

      } else {

        if(nematode==4) {
          #C oncophora

          dev.1 = pmax(0, -0.0401 + 0.00821 * temp)
```

```
          dev.1 = pmin(1, dev.1)
          mu.1 = pmin(1, exp(-4.38278 - 0.1064 * temp + 0.0054 * (temp^2))
)
          mu.2 = pmin(1, exp(-4.38278 - 0.1064 * temp + 0.0054 * (temp^2))
)
          mu.4 = pmin(1, exp(-5.822 - 0.1749 * temp + 0.0070 * (temp^2) +
0.00002 * (temp^3)))
          mu.3 = pmin(1, exp(-4.726 - 0.204 * temp + 0.0044 * (temp^2) + 0
.00009 * (temp^3)))
          mu.5 = mu.3
          h.mig = ifelse(precip>=6, pmax(0, pmin(1, exp(-3.35002 + 0.05559
*precip))),0)
          v.mig = (pmax(0, exp(-5.48240 + 0.45392*temp - 0.01252*(temp^2))
*0.459)) ##vmig updated with recovery rate.

          egg.correction = rep(1, length(temp))

          dev.2 = 0.04077336
          max.mu6 = 0.12 # high pre-adult mortality in Verschave thesis ht
tps://biblio.ugent.be/publication/6986324
          min.mu6 = 0.044 # mean pa mortality in Verschave thesis
          mu7 = 0.002
          min.mu8 = 0.04 # mean Verschave thesis
          max.mu8 = 0.12 # high Verschave thesis
          min.h = 0
          max.h = 0.06
          min.lambda = 6.44
          max.lambda = 7.30
          rho.response = 1.316e-4
          sigma.decay = 0.002
          pAf = 0.5

          devsuccess = as.numeric(na.ma(ma(dev.1, order = 7,centre = FALSE
), k = 7))
          hypobiosis = max.h - ((max.h-min.h)/(max(devsuccess, na.rm = TRU
E)))*(devsuccess) # (max(devsuccess, na.rm = TRUE)-min(devsuccess, na.rm =
TRUE)))*(devsuccess-min(devsuccess, na.rm = TRUE)) #max.h - ((max.h-min.h)
/(max(photoperiod)-min(photoperiod)))*(photoperiod-min(photoperiod))
          resume = (1/(max(devsuccess, na.rm = TRUE)-min(devsuccess, na.rm
= TRUE)))*(devsuccess-min(devsuccess, na.rm = TRUE))

          print('Cooperia parameters sourced')

          (sapply(ls(),function(x)get(x),simplify=F,USE.NAMES=T))

        }
      }
    }
  }
}
```

S2 Simulation Set 2

S2.1 Overview

Simulation Set 2 concerned the investigation of parameters which may impact the evolutionary-epidemiological of GIN infections over time. The focus of this simulation set was to understand the factors which drive transmission and resistance.

S2.2 Calibration of Transmission Intensity

For simulation sets 2 (“Evolutionary epidemiological considerations of anthelmintic treatments”) and 3 (“Evaluating Anthelmintic Resistance Management Strategies”) in the main manuscript, we needed to generate simulations which covered a range of plausible transmission settings and transmission intensities. This is because there could be some prior expectation that transmission intensity may interact with both treatment efficacy (e.g., reinfection) and resistance management (e.g., the comparative size of refugia populations). It was therefore needed to explore the implication of different transmission levels.

We aimed to have two broad transmission settings, termed a “moderate” and “high” transmission setting. The aim was for the “moderate” transmission intensity simulations to have a mean (across the sampled locations) peak FEC in the second year in the absence of treatment or other interventions of ~700epg and for the “high” setting this was ~1250epg. It is important to note that because different climate settings (locations) also impact transmission through the population dynamics of the GIN populations, the actual values achieved in any an individual simulation are likely different. However, they can still be used to describe qualitatively different levels of transmission, such that for each climate setting there is a “moderate” and “high” transmission scenario. It is just the actual levels achieved may be either above or below the desired values.

In the simulation code this was set by defining the starting number of L3 free-living larvae on pasture, and was the varied until the mean peak FEC (with 100 geographic locations and climatic conditions used) in the second year hit the desired level when using different numbers of input L3. Figure S2.1 shows the result from this calibration, and we therefore used 65,000 L3p as the input for “moderate” transmission and 160,000 L3p as the input value for the “high” transmission setting.

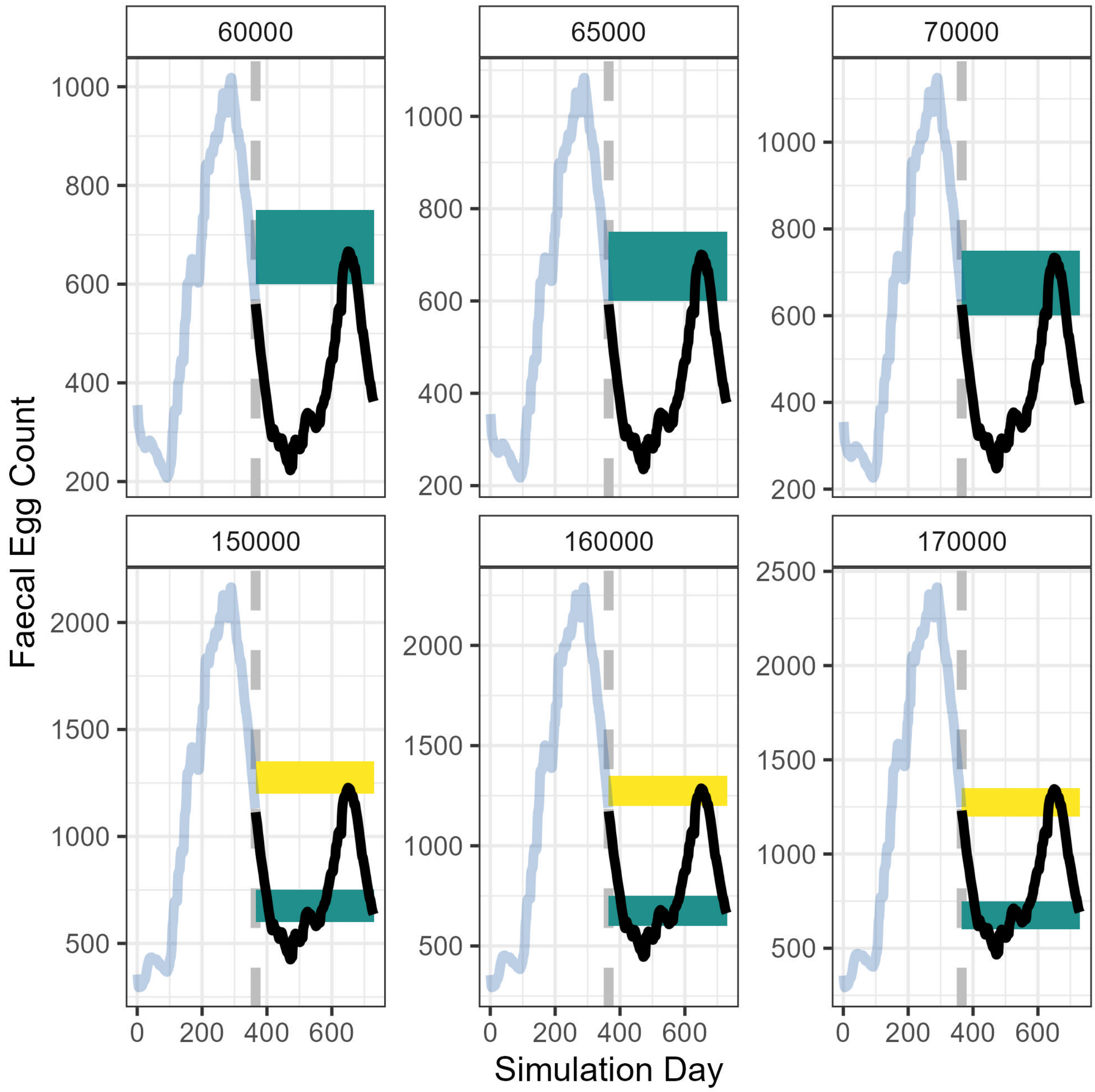

Fig. S2.1. Calibration of the Initial Larval Pasture Seeding for the Simulations to Give Required Target Faecal Egg Count for Varying Transmission Intensity. Each panel represents the different input number of L3 pasture input into the model. The light blue line is the first year of the simulation and the black line is the second year of the simulation, where the aim is to obtain the target faecal egg count for the simulations. This plotted simulation line is the mean value of 100 geographic locations to represent a variety of climatic conditions which further impacts the dynamics of the nematode populations. The yellow rectangle indicates the range for which a "high" transmission setting would be defined, and the green rectangle for a "moderate" transmission setting.

S2.3 Calculating anthelmintic drug parasite killing efficacy given drug decay:

In the simulations where drug decay was included there was a need to include a drug efficacy decay curve. Given that different drugs are given at different concentrations, and the drug concentration to parasite killing efficacy will vary between drug formulation, rather than tracking drug decay as the amount of the physical drug itself, the efficacy of the drug itself was used. This allows for a far more generalisable system, and further allows for an agnostic approach to precise drug formulations. While there are many potential ways to include drug decay, we consider a simplistic method of exponential decay:

$$\kappa_t^{genotype} = \kappa_0^{genotype} * \exp(-(\tau_t - \tau_d)^2 \gamma_d), \text{ when } \tau_t > \tau_d$$

Equation 10

Where $\kappa_0^{genotype}$ is the killing efficacy of the drug of the specified genotype when at the target drug concentration in the livestock, that is the registered therapeutic dose. τ_t is the number of days since the anthelmintic drug was administered. τ_d is the number of days after administration the drug begins to decay. The value γ_d is the decay rate, which is 0.005 for simulations with decay, and 1 for simulations without decay, which can be used to give the drug efficacy and decay profiles given in Figure S2.2, and are those used in simulation set 1.

S2.4 Conditions varied for sensitivity analysis

**Table S2.1 Parameters and conditions explored in Simulation Set 1.**	
**Condition**	**Values Explored**
Initial resistance allele frequency	1%, 10%
Initial SS efficacy	0.99, 0.9
Initial RR efficacy	0.01, 0.9
Dominance	0.25, 0.5, 0.75
Drug decay	Absent, present*
Transmission intensity	High, moderate**
Livestock refugia size	0%, 10%, 20%
*Detailed in the “Calculating anthelmintic drug parasite killing efficacy given drug decay” section, and Figure S2.2<br>**Detailed in the “Calibration of Transmission Intensity” section, and Figure S2.1	

S2.5 Supplementary Analysis

Given the wide range of parameter values explored (288 total permutations, Table S2.1), we only presented a subset of this data in the main manuscript. In this supplementary analysis, we further consider all the permutations of parameters investigated. But note the main qualitative conclusions identified in the main manuscript are unchanged in this further exploration. Nevertheless, given the current uncertainty with how different parameter values may interact, it was still prudent to explore these additional permutations, and therefore provides further robustness of the presented conclusions.

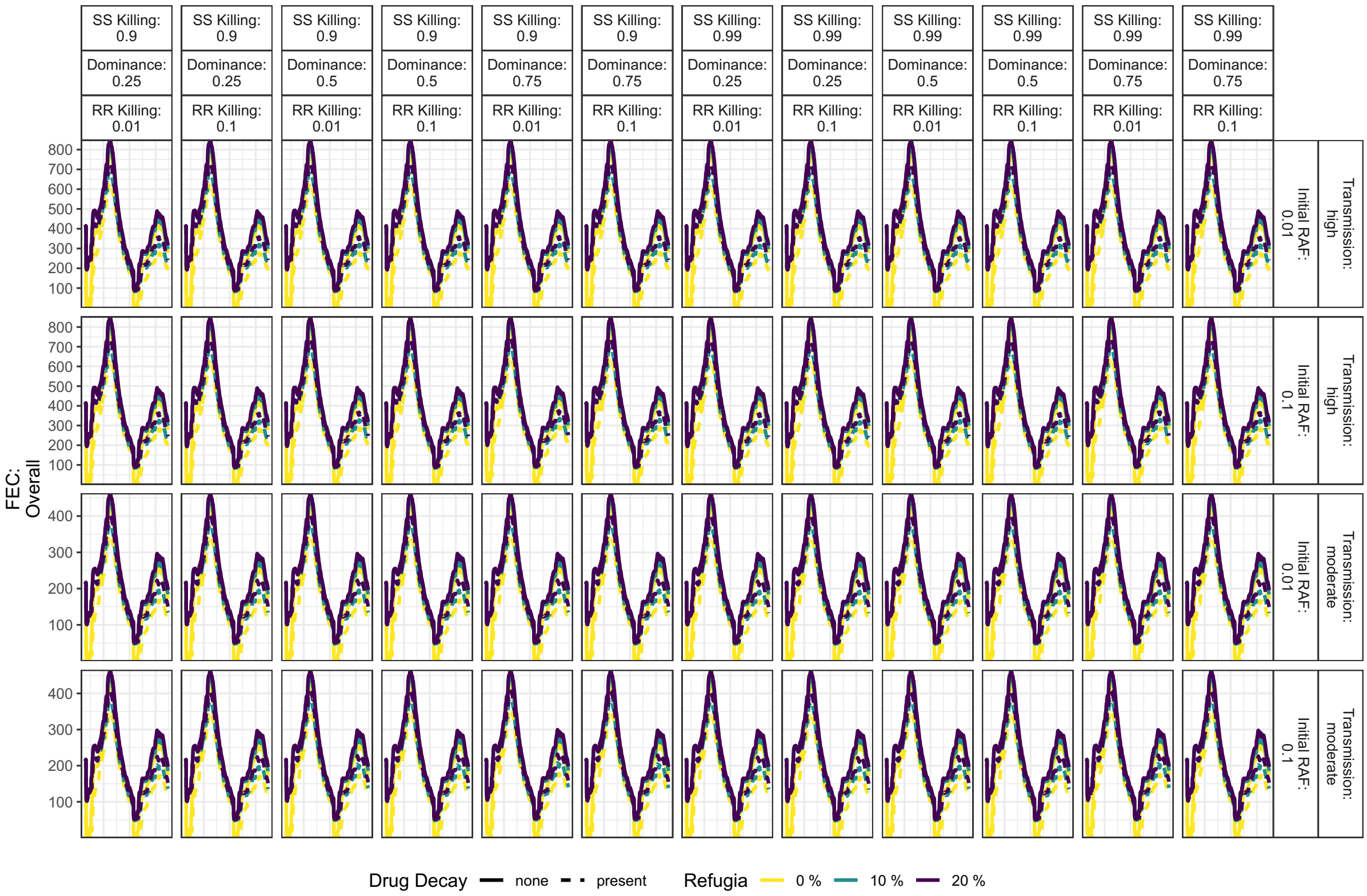

Fig. S2.3. Full Results for Simulation Set 2 Overall Faecal Egg Count, Including All Input Parameter Permutations. The plot is stratified left-right by the resistance phenotypes of the SS, RS and RR genotypes; where the RS phenotype is dependent on the dominance of resistance. The plot is stratified top-bottom by the overall level of transmission and the initial resistance allele frequency (RAF) at the start of the simulation. The outcome (y axis) is the overall faecal egg count for all livestock (cohort 1 and cohort 2).

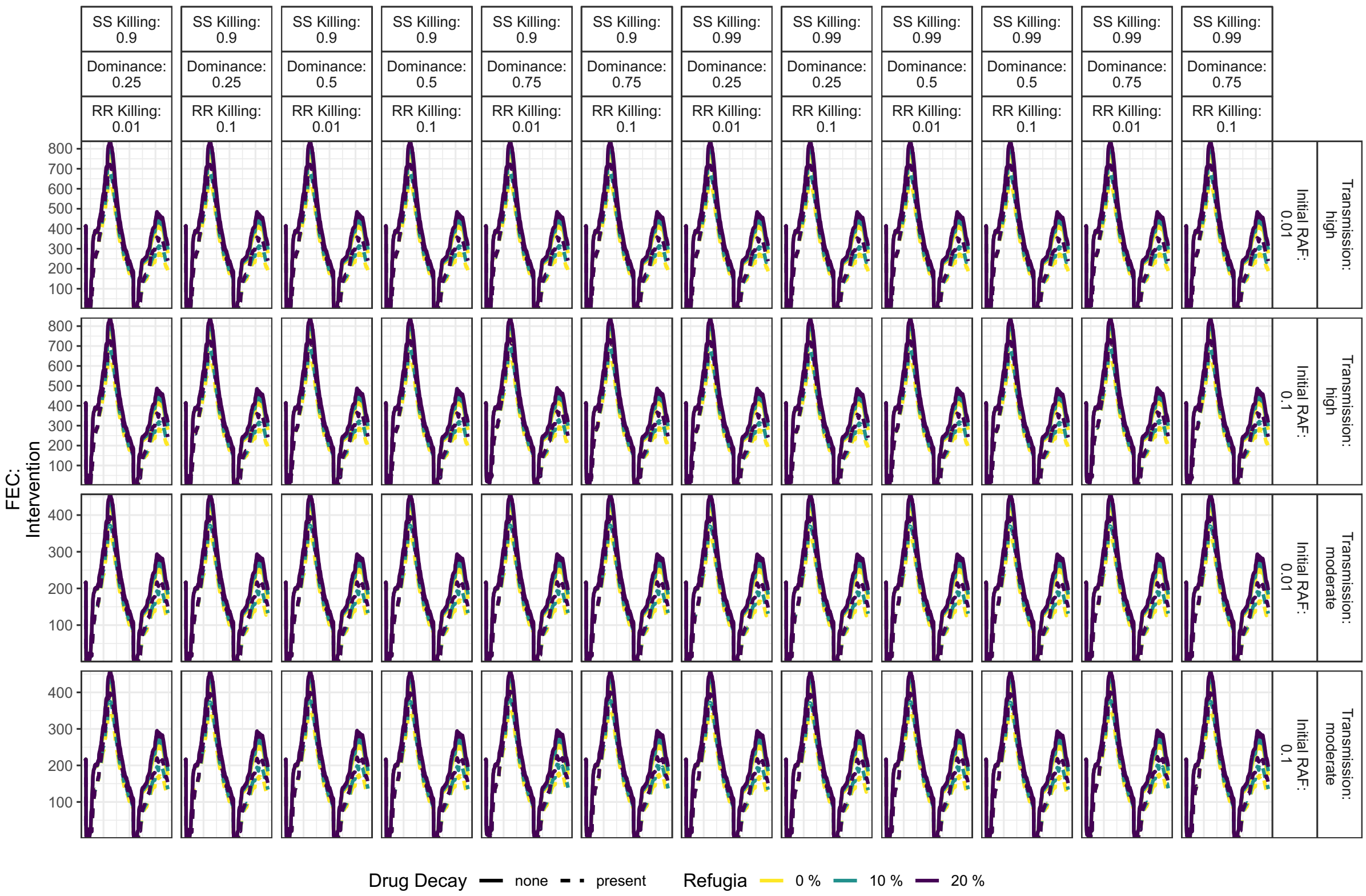

Fig. S2.4. Full Results for Simulation Set 2 Faecal Egg Count for Intervention Livestock, Including All Input Parameter Permutations. The plot is stratified left-right by the resistance phenotypes of the SS, RS and RR genotypes; where the RS phenotype is dependent on the dominance of resistance. The plot is stratified top-bottom by the overall level of transmission and the initial resistance allele frequency (RAF) at the start of the simulation. The outcome (y axis) is the overall faecal egg count for only the treated "intervention" livestock (cohort 1).

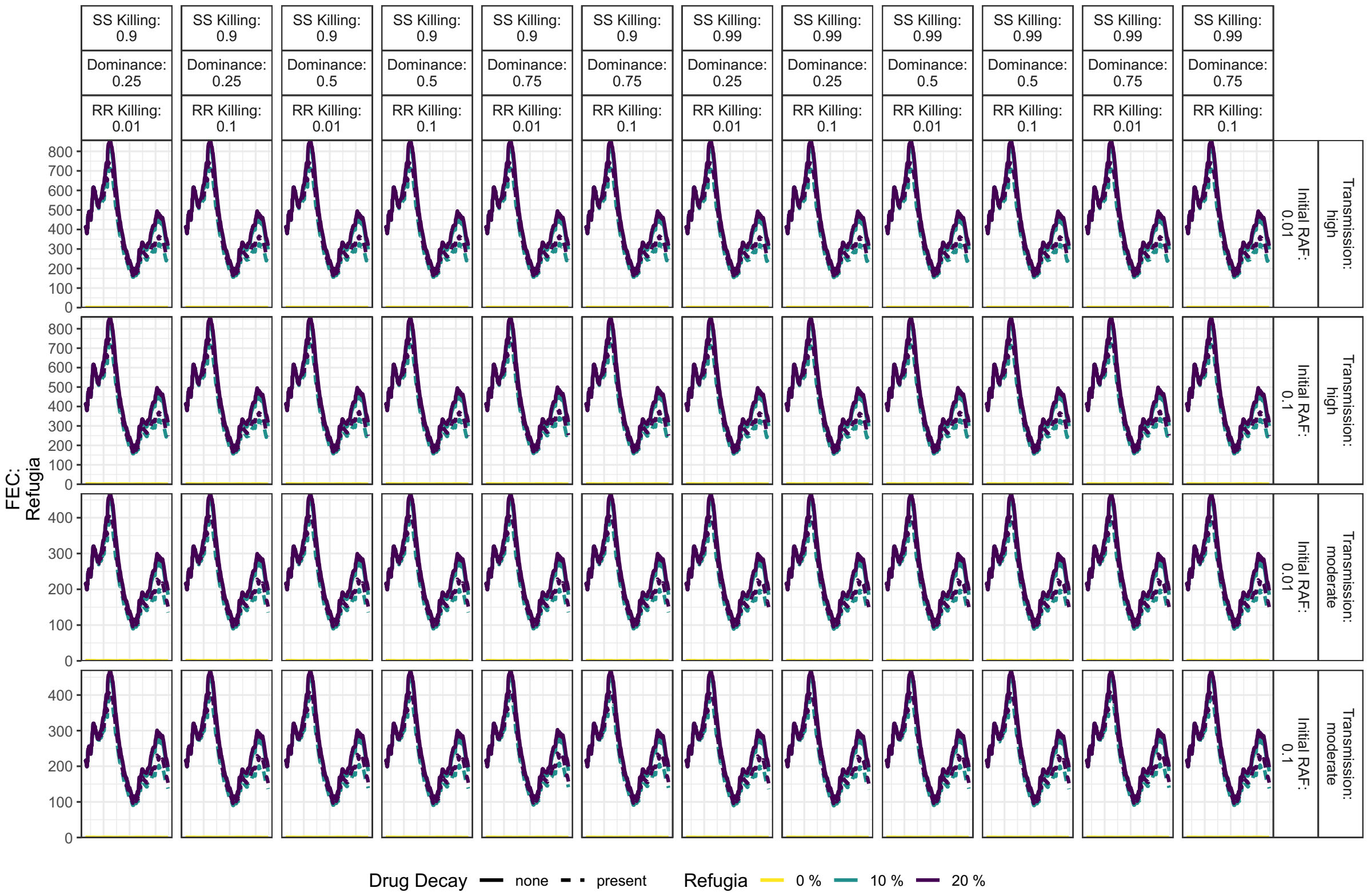

Fig. S2.5. Full Results for Simulation Set 2 Faecal Egg Count for Refugia Livestock, Including All Input Parameter Permutations. The plot is stratified left-right by the resistance phenotypes of the SS, RS and RR genotypes; where the RS phenotype is dependent on the dominance of resistance. The plot is stratified top-bottom by the overall level of transmission and the initial resistance allele frequency (RAF) at the start of the simulation. The outcome (y axis) is the overall faecal egg count for only the untreated “refugia” livestock (cohort 2).

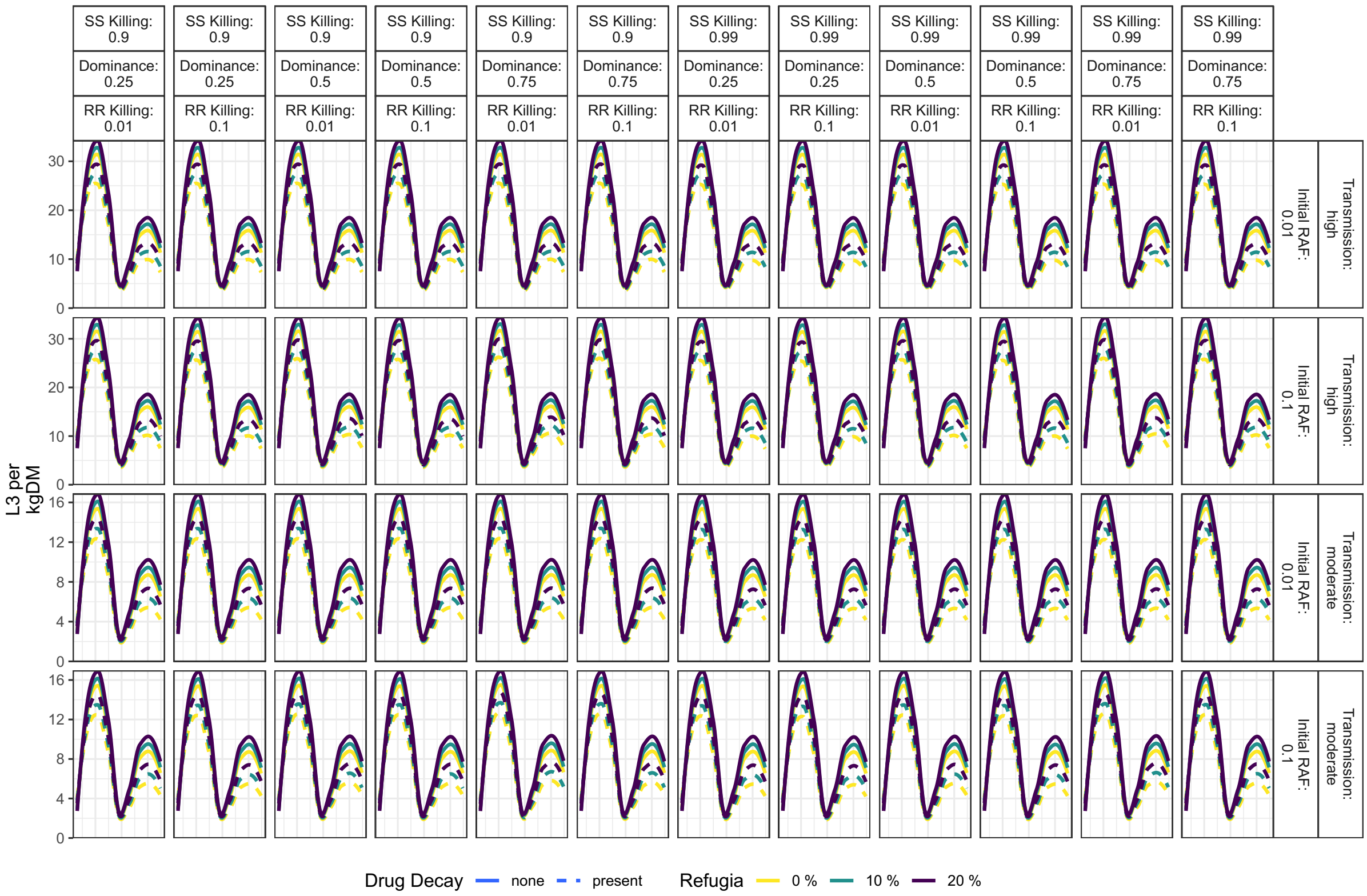

Fig. S2.6. Full Results for Simulation Set 2 Pasture Larval Counts, Including All Input Parameter Permutations. The plot is stratified left-right by the resistance phenotypes of the SS, RS and RR genotypes; where the RS phenotype is dependent on the dominance of resistance. The plot is stratified top-bottom by the overall level of transmission and the initial resistance allele frequency (RAF) at the start of the simulation. The outcome (y axis) is the L3 per kg of dry matter, which can be considered a measure of transmission intensity.

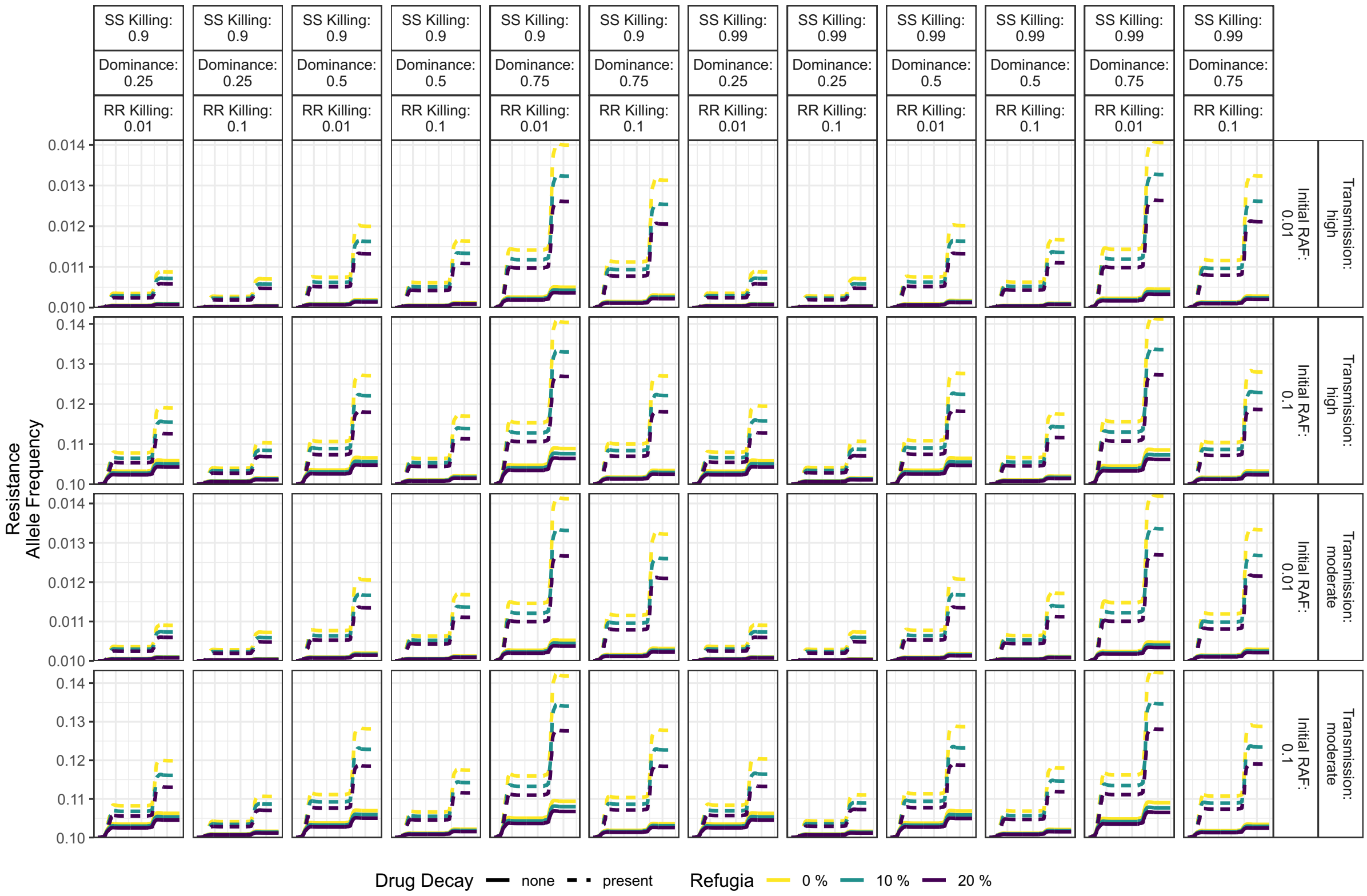

Fig. S2.7. Full Results for Simulation Set 2 Resistance Allele Frequency, Including All Input Parameter Permutations. The plot is stratified left-right by the resistance phenotypes of the SS, RS and RR genotypes; where the RS phenotype is dependent on the dominance of resistance. The plot is stratified top-bottom by the overall level of transmission and the initial resistance allele frequency (RAF) at the start of the simulation. The outcome (y axis) is the resistance allele frequency, which can be considered a measure of resistance management.

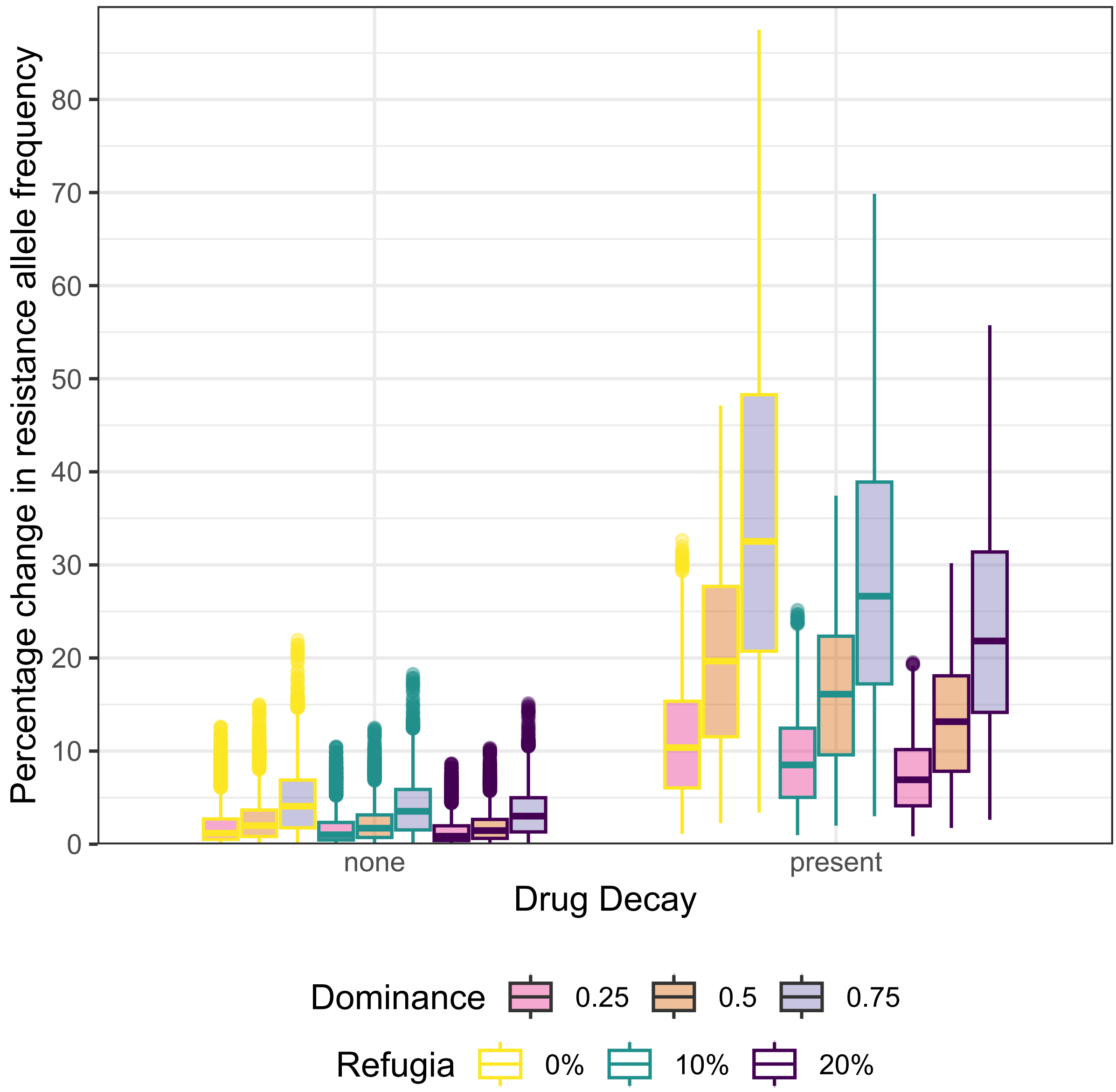

Fig. S2.8. Percentage Change in the Resistance Allele Frequency at the End of the Simulation (Day = 1086).

S3 Simulation Set 3

S3.1 Overview

Simulation set 3 ("Evaluating Anthelmintic Resistance Management Strategies") involved to direct comparison of different ARM strategies, with the aim to identify the broad level performance of ARM strategies. It should be noted that there is much uncertainty with modelling resistance management (Hastings, 2001) and so only qualitative inferences should be made. Modelling can be used to explore otherwise complicated strategies (e.g., cohort mosaics), where a theoretical advantage would be needed to justify further (expensive and time consuming) empirical investigation.

S3.2 Detailed Parameter Descriptions

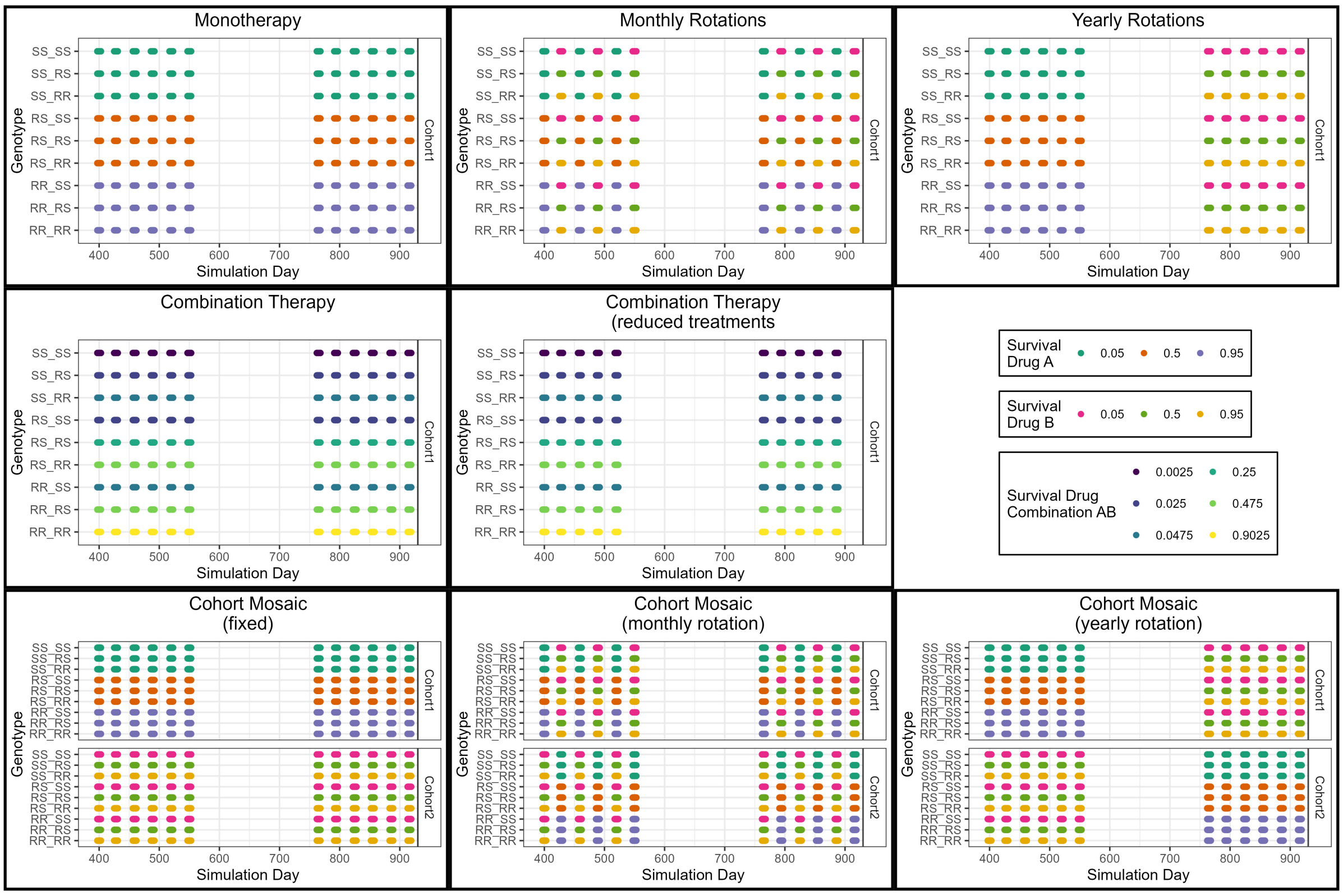

Fig. S3.1. Conceptualisation of the ARM Strategies with Survival Probabilities. Each panel shows the survival for the genotypes at the time points when given the designated drug treatments. The colours indicate the survival probability for the genotype at the specified time to the anthelmintic drug treatment which will be given (as detailed in the figure legend panel).

S3.3 Conditions varied for sensitivity analysis

Table S2.1 Parameters and conditions explored in Simulation Set 2.	
**Condition**	**Values Explored**
Initial resistance allele frequency: Drug 1	1%, 10%
Initial resistance allele frequency: Drug 2	0.99, 0.9
Strategy	Monotherapy, Monthly pharmacological rotation, Yearly pharmacological rotation, Fixed cohort mosaic, Monthly cohort mosaic, Yearly cohort mosaic, Combinations, Combinations with reduced coverage, Combinations with reduced frequency

Table S2.1 Parameters fixed in Simulation Set 2.	
**Condition**	**Values Explored**
Parasite killing efficacy of each drug against homozygous susceptible	95%
Parasite killing efficacy of each drug against homozygous resistance	5%
Dominance of Resistance	0.5
Transmission intensity	
Refugia Size	20% (30% in Combinations with reduced coverage)
Number of treatments per year	6 (5 in Combinations with reduced frequency)

S3.4 Supplementary Analysis

Figure S3.2 shows the absolute faecal egg counts for the livestock populations. A clear indication here is that, regardless of the level of resistance or ARM strategy, the overall epidemiological difference is small. However these differences do become larger when there are higher levels of resistance.

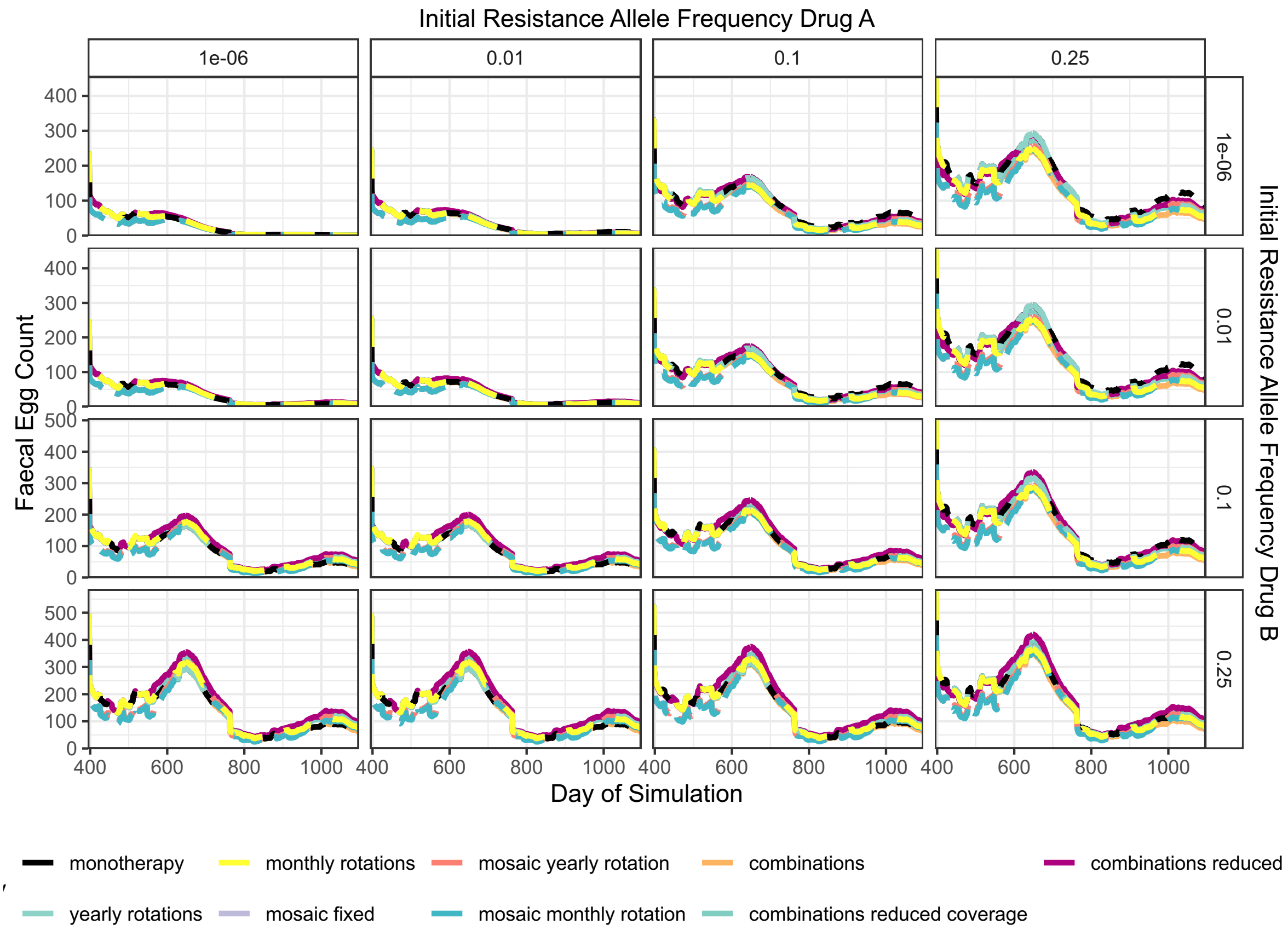

Fig. S3.2. Simulation Set 3: Faecal Egg Count in Response to Anthelmintic Drug Treatments. The y axis is the faecal egg count. The long-dash lines indicate a rotation strategy, short-dash lines a cohort mosaic strategy, and solid lines indicate a combination therapy strategy. The colour of the lines indicates the specific strategy. The x-axis is the simulation day and is restricted to not include the 1-year burn-in which is the same across all strategies. Each line is the mean of 100 simulations, covering a range of geographic locations.

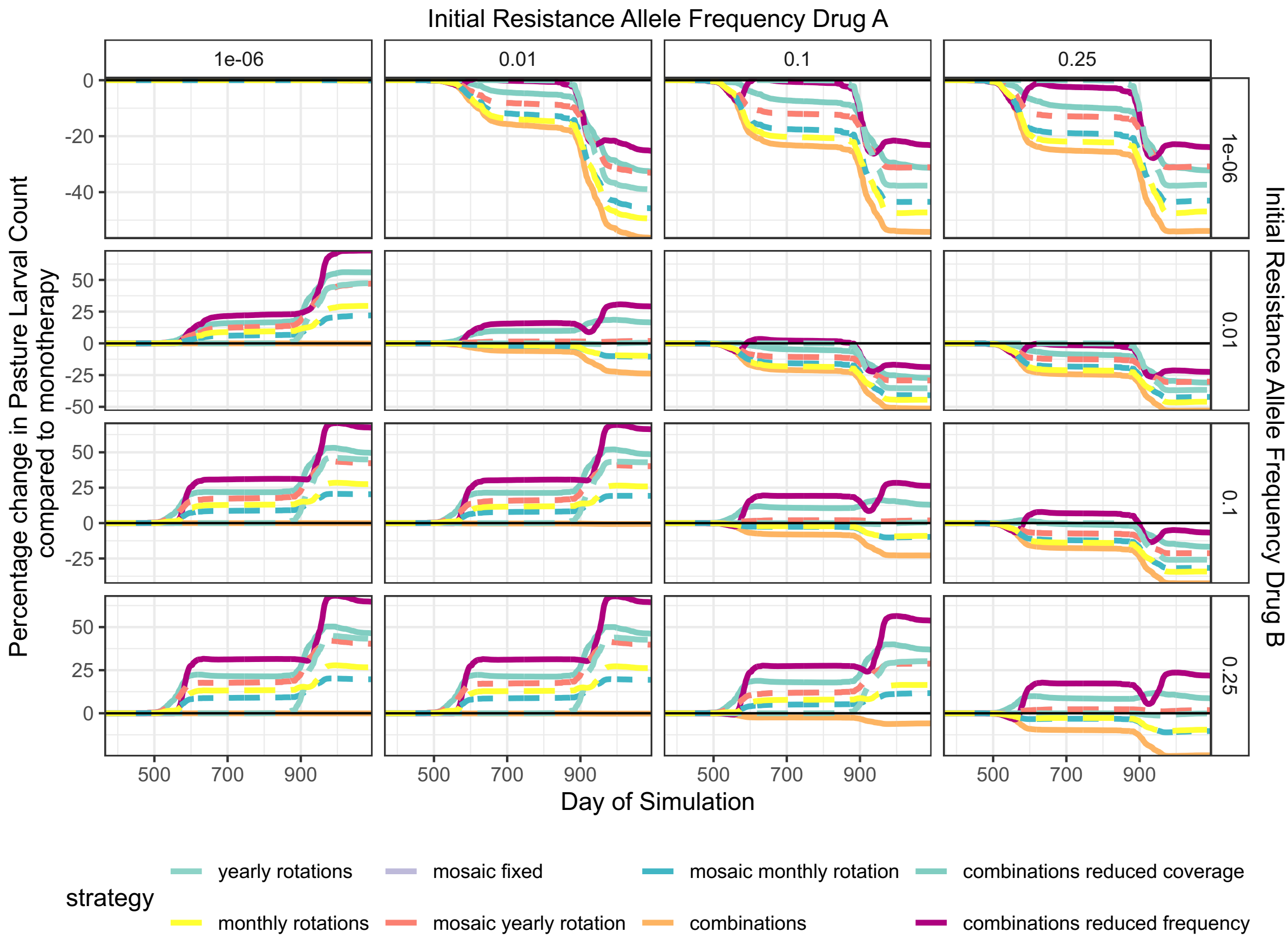

Fig. S3.3. Simulation Set 3: Percentage Change in Larval Pasture Counts. The y axis is the percentage change in the larval pasture count, as calculated using Equation 1 in the main manuscript. The horizontal black line (at zero) represents the no difference between strategies. Values below the black line indicates the proposed strategy performed better, and values above performed worse than the Monotherapy comparator. The long-dash lines indicate a rotation strategy, short-dash lines a cohort mosaic strategy, and solid lines indicate a combination therapy strategy. The colour of the lines indicates the specific strategy. The x-axis is the simulation day and is restricted to not include the 1-year burn-in which is the same across all strategies. Each line is the mean of 100 simulations, covering a range of geographic locations.

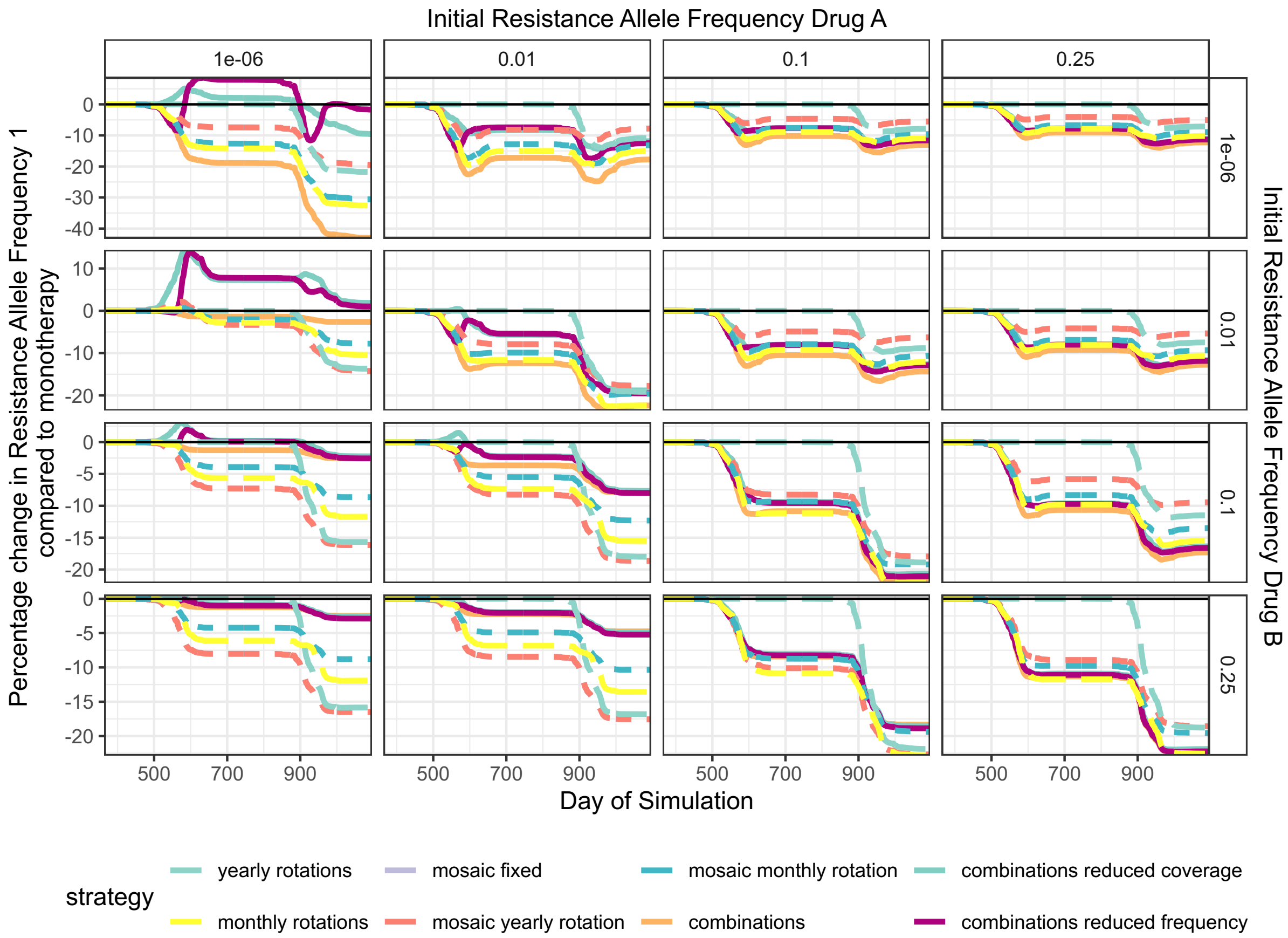

Fig. S3.4. Simulation Set 3: Percentage Change in the Resistance Allele Frequency To Drug 1. The y axis is the percentage change in the resistance allele frequency for Drug 1, as calculated using Equation 1 in the main manuscript. Note, Drug 1 is the drug used in the monotherapy simulations and Drug 2 is not deployed in the monotherapy simulations. The horizontal black line (at zero) represents the no difference between strategies. Values below the black line indicates the proposed strategy performed better, and values above performed worse than the Monotherapy comparator. The long-dash lines indicate a rotation strategy, short-dash lines a cohort mosaic strategy, and solid lines indicate a combination therapy strategy. The colour of the lines indicates the specific strategy. The x-axis is the simulation day and is restricted to not include the 1-year burn-in which is the same across all strategies. Each line is the mean of 100 simulations, covering a range of geographic locations.

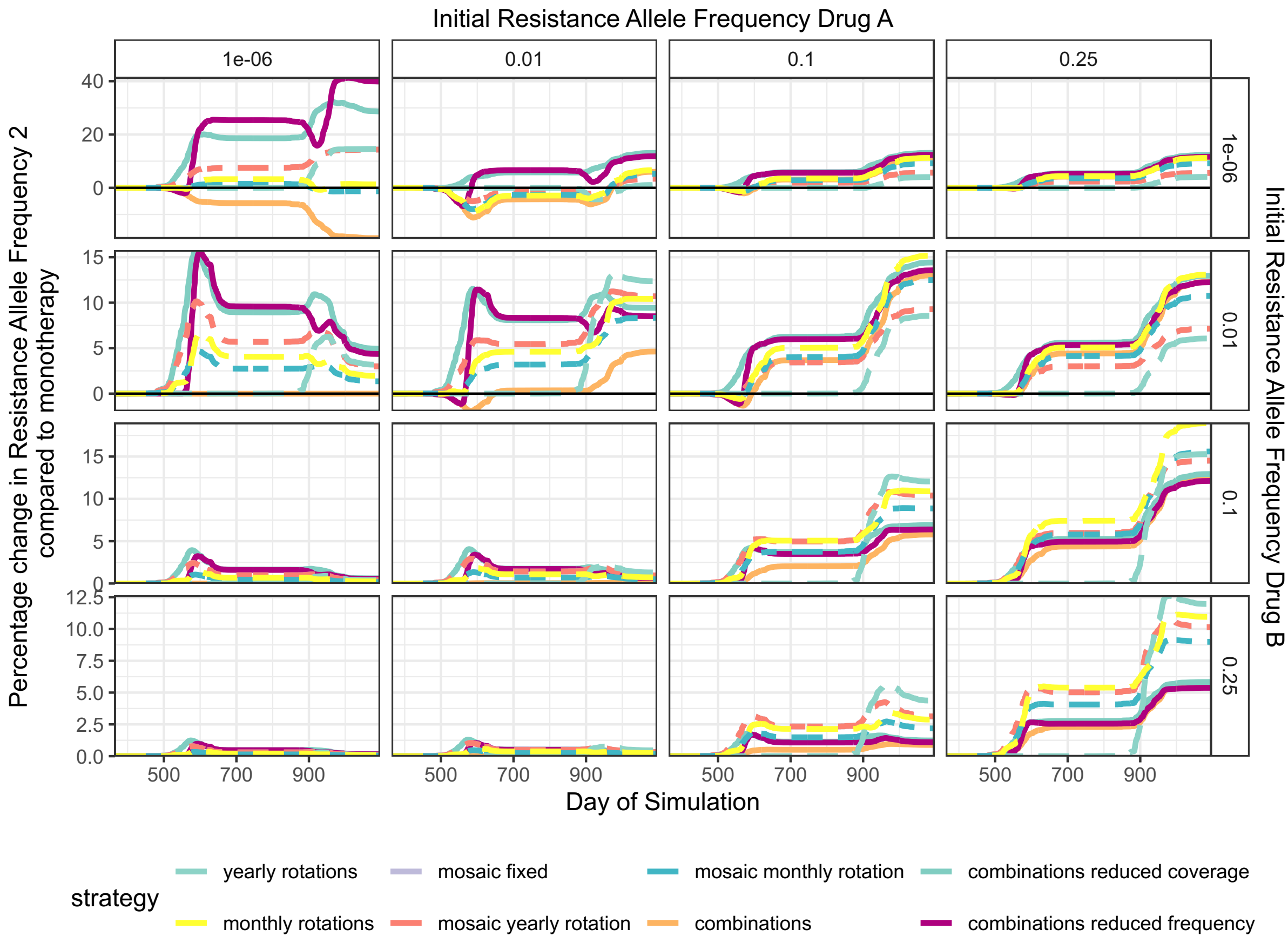

Fig. S3.5. Simulation Set 3: Percentage Change in the Resistance Allele Frequency to Drug 2. The y axis is the percentage change in the resistance allele frequency for Drug 2, as calculated using Equation 1 in the main manuscript. Note, Drug 1 is the drug used in the monotherapy simulations, and Drug 2 is not deployed in the monotherapy simulations. The horizontal black line (at zero) represents the no difference between strategies. Values below the black line indicates the proposed strategy performed better, and values above performed worse than the Monotherapy comparator. The long-dash lines indicate a rotation strategy, short-dash lines a cohort mosaic strategy, and solid lines indicate a combination therapy strategy. The colour of the lines indicates the specific strategy. The x-axis is the simulation day and is restricted to not include the 1-year burn-in which is the same across all strategies. Each line is the mean of 100 simulations, covering a range of geographic locations.

S4 GLOWORM Simulation Framework Showcase

S4.1 Overview

Given the increase in the complexity of the GLOWORM simulation framework, it becomes beneficial to display the technical ability of the current simulation; especially as a proof of principal of what the simulation framework can be used to for. These simulations are to present only the technical simulation capability, and ***conclusions regarding anthelmintic deployment practices must not be drawn from these presented examples***. The simulations in these examples deliberately use arbitrary values, which we intentionally do not report, to showcase the model features. Each example simulation is presented to showcase the model capability, and should not be used to interpret the effectiveness of any worm control practices.

S4.2 Example Simulations

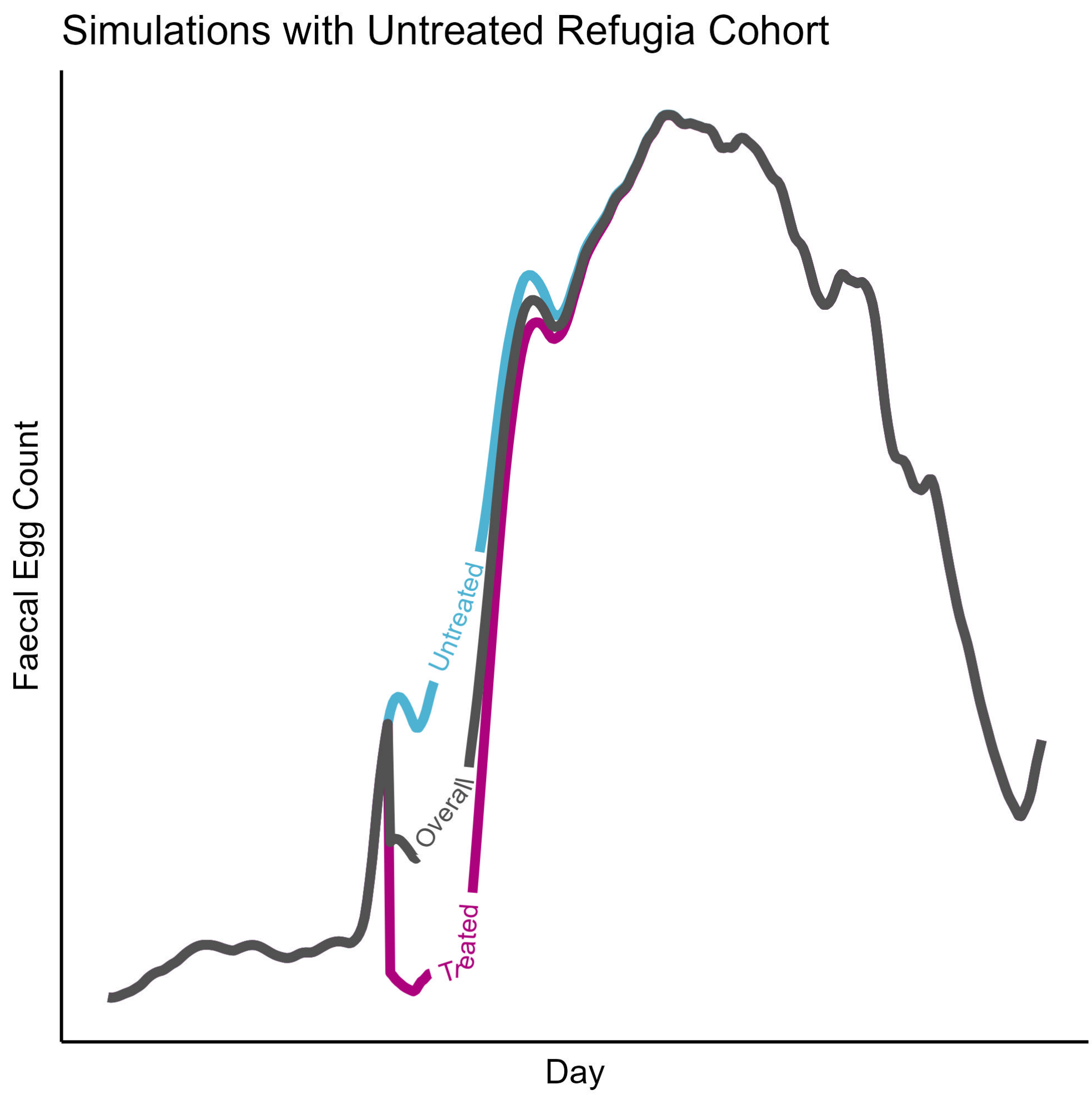

Fig. S4.1. Untreated Livestock Refugia Inclusion of explicit refugia populations. As each cohort in the model simulations can be given unique treatments, the inclusion of an explicitly modelled untreated livestock refugia can be included. The use case for this will be for evaluating how the relative size of refugia populations influences resistance frequency, but also understanding the disease control implications of leaving portions of the livestock population untreated.

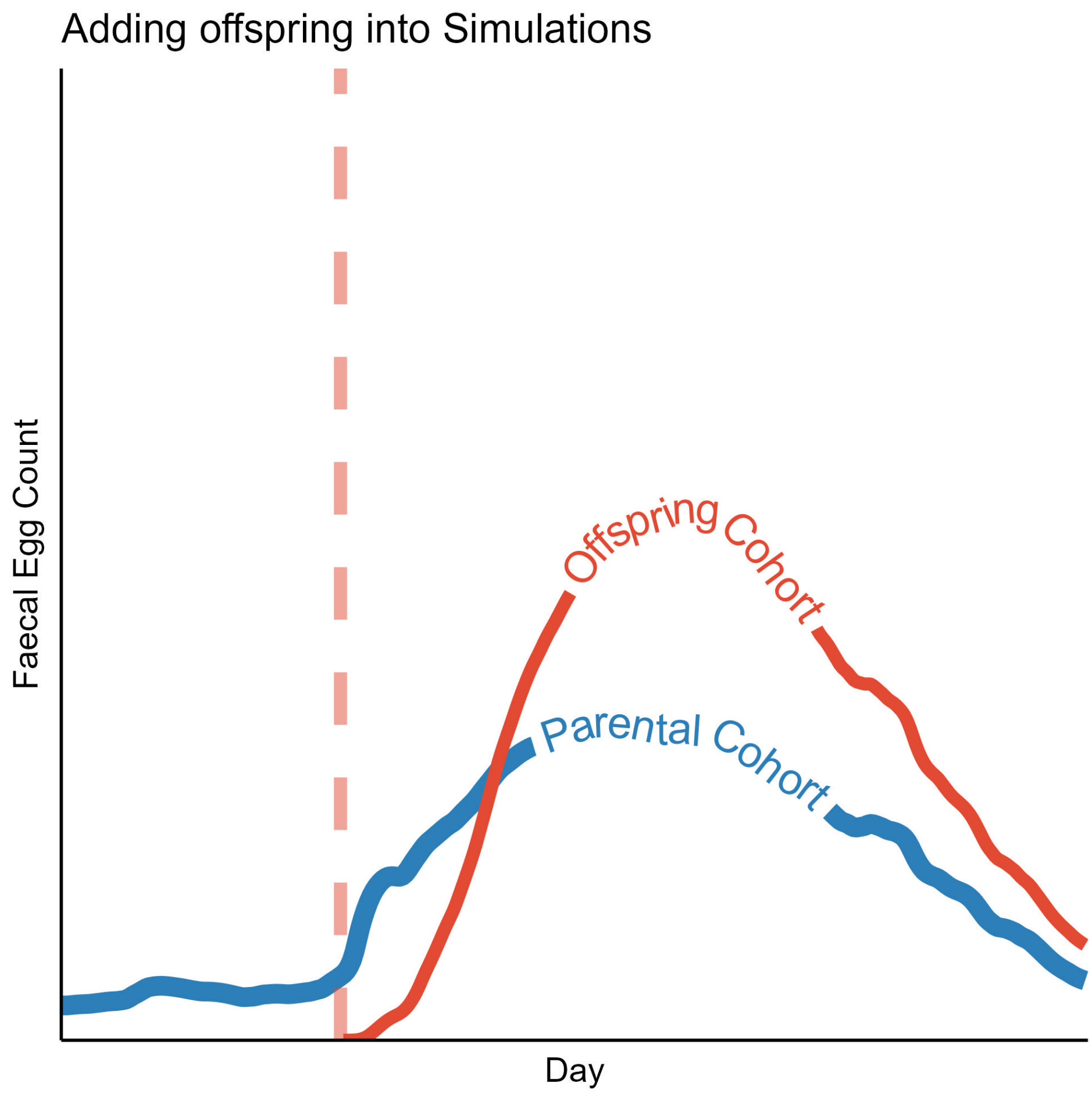

Fig. S4.2. Adding in Parent-Offspring Livestock into the Simulations. The simulations can used to add in a new "cohort" which is the offspring of another "parental" cohort of in the simulations. The applicability of this can therefore be to look at the implication of the periparturient rise in faecal egg count which is observed to happen to parental ewes at lambing time. The offspring cohort can then have a different weight, which then relates to dry matter intake and faecal production.

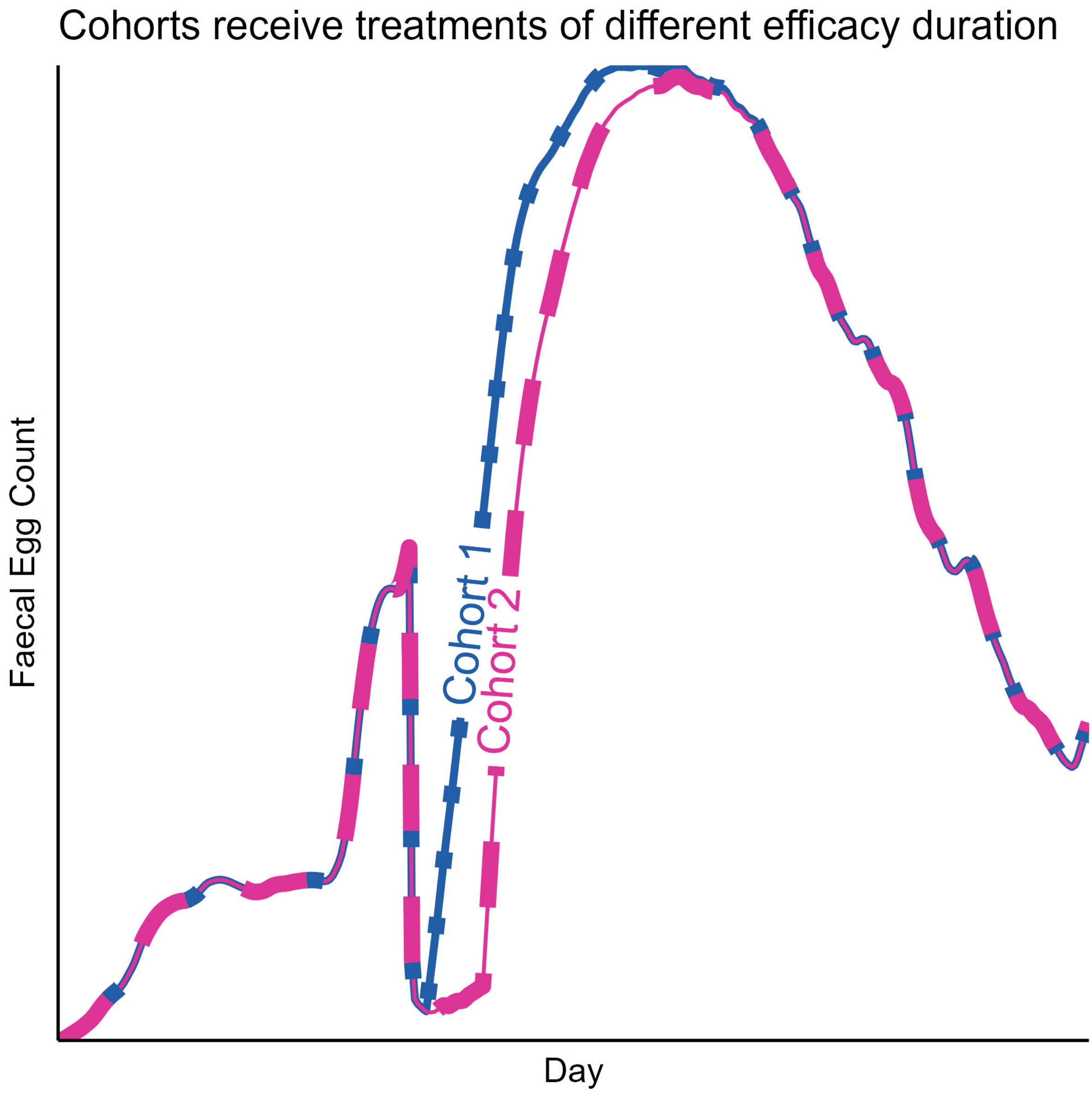

Fig. S4.3. Livestock Cohorts Receive the Same Treatment, but has Different Efficacy Durations. Each cohort can be given the same drug formulation, however to account for the heterogeneity in drug efficacies between livestock the drugs can be given different efficacy profiles for each cohort. The use case here is therefore to explore how treatment heterogeneities impact the spread of disease and resistance.

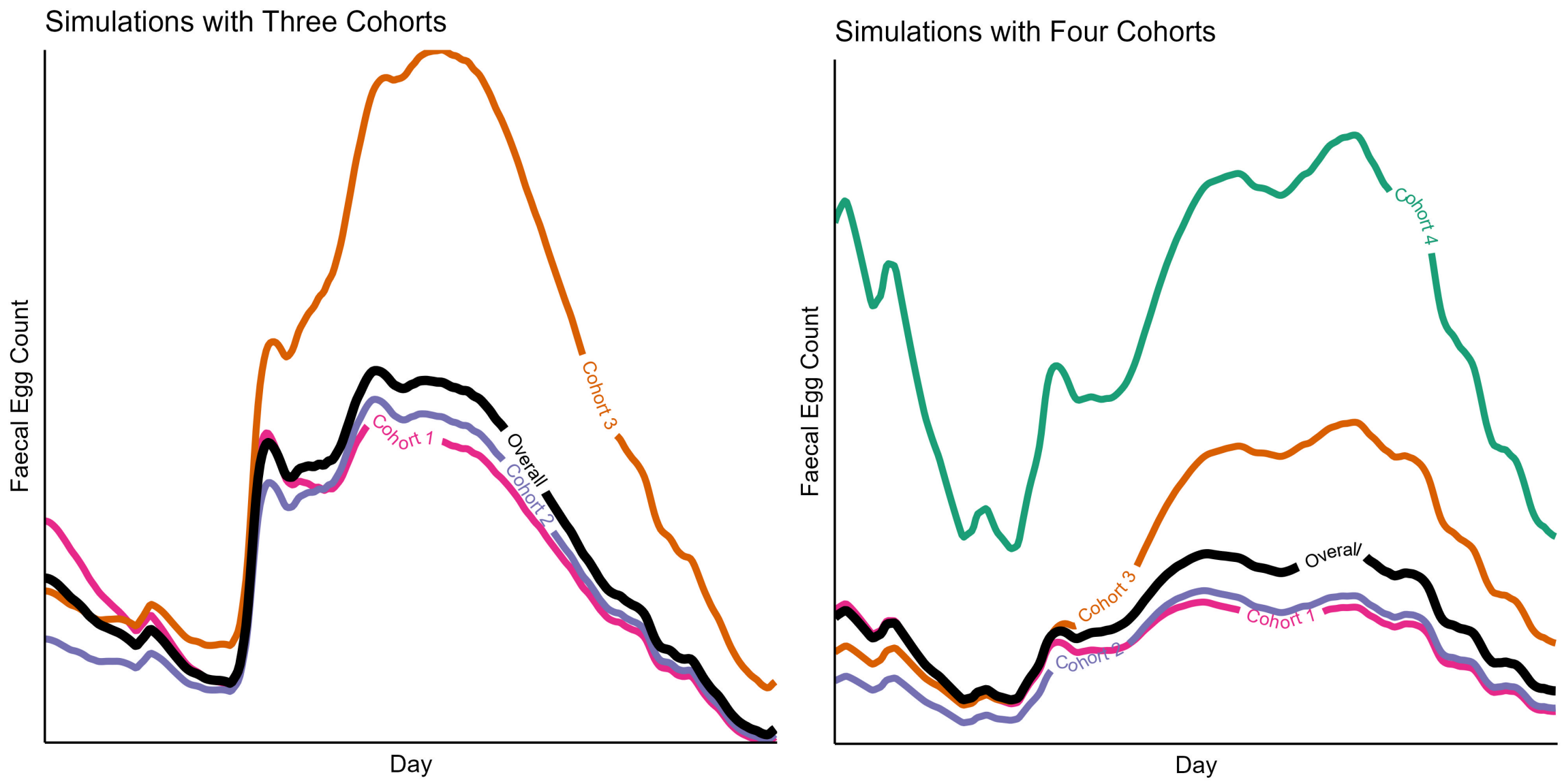

Fig. S4.4. Simulations with Multiple Livestock Cohorts. Simulations can be conducted with up to four separate livestock cohorts. Who can each be given unique ages (bodyweights) and dry matter intakes. The panel on the left shows a simulation with three cohorts, and the panel on the right shows the simulation with four cohorts.

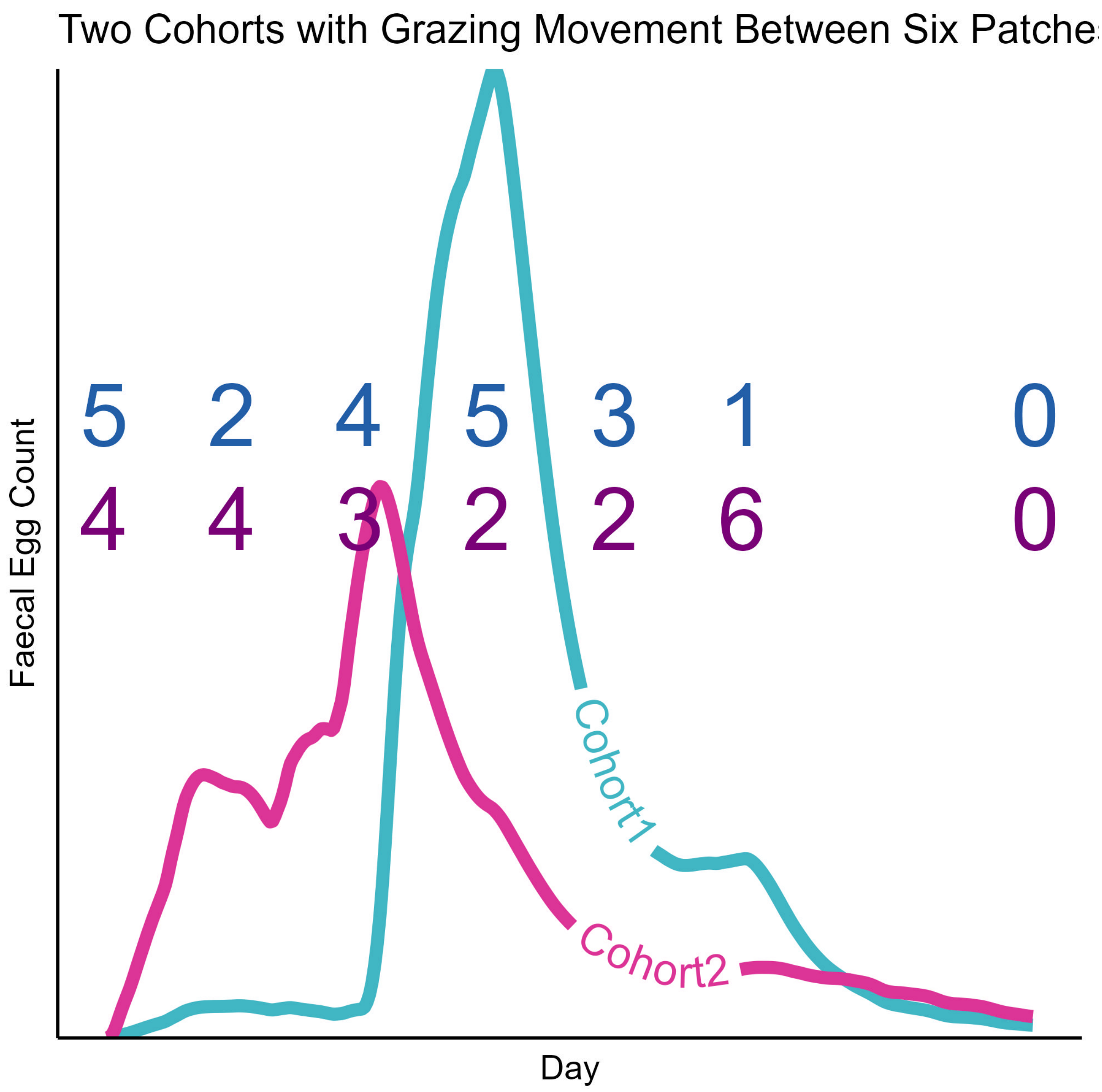

Fig. S4.5. Two Livestock Cohorts with Different Rotational Grazing Movements. Each cohort can also be assigned a unique pattern of movements between patches. Here the location of the cohort is distinguished by the number, which shows when they moved the patch with the number identification number.

S5 Model Validation

S5.1 Model Validation Overview

The purpose of this model validation is to understand how the GLOWORM model framework performs against real-world empirical data.

As part of this modelling exercise, we attempt to validate our simulation platform against published empirical results from field trials. This helps us to better understand the performance of our model in relation to trial data.

We perform our model validation against three data sets.

1. Stear study (Stear et al., 2006)
2. Sargison study (Sargison et al., 2012)
3. Waller study (Waller and Thomas, 1975)

We would note that validating against trial data does have several challenges. First, we are limited to validating against the reported findings from trials. Trials are expensive and time-consuming to conduct, which unfortunately limits the amount (and variety) of data which is collected. In general, the primary outcome which we are validating against is the reported faecal egg counts from these studies.

Uncertainty in values from published literature:

1. Exact geolocation. This impacts the latitude and longitude for the extraction of the climate data (rainfall and temperature).
2. Pasture contamination.
3. Parasite level in adult ewe populations.
4. Stocking rates and livestock rearing density.
5. Treatment timings (exact); and exact treatment efficacies.

The SCOPE trial (Rose Vineer et al., 2025) hopes to rectify some of the issues with validating and testing models against field trials. Whereby mathematical modellers are directly integrated into the trial team, ensuring trial design is designed around parameters which can be included into a mathematical modelling framework.

S5.2 Validation against Stear Study

We attempt to validate our simulations against the results in Figure 4 in (Stear et al., 2006). For simplicity we refer to this study as the Stear study. This data is the mean from two study sites replicated over multiple years. We summarise the conditions in

Table S5.1 noting in particular any key time-frames or values with which we use to set up our simulations. The data from both farm locations was used to produce, but we note that the details of Farm 1 (“west central Scotland”) are better detailed, and therefore only attempt to replicate Farm 1. We simulate two cohorts, a ewe cohort and a lamb cohort.

**Table S5.1 Validation of GLOWORM using the Stear Study**		
Study Aspect	Stear Study	GLOWORM Simulations
Parasite species	*Teladorsagia circumcincta*, *Cooperia* spp., *Trichostrongylus vitrinus*, *Trichostrongylus axei*, and *Haemonchus contortus.*	We assume a single species infection with the parasite *T. circumcincta*.
Geolocation and weather	The location of the farm is described as being in “west central Scotland”.	We assume the study was in an area bounded by the latitude and longitude coordinates of: latitude: min = 55.69, max = 55.81; and longitude min = -4.55, max = -4.39. We sample locations in the latitude and longitude direction at 0.02 intervals, giving a total of 63 sampled geolocations. Extract the weather data for these coordinates using the openmeteo R package (Pisel, 2023)
Timeframe	April-October for years 1990 to 1995	April-October for years 1990 to 1995
Lambing	Lambing occurred during a 3-week period in April-May.	Lambing occurs as a single event where all lambs are born (creation of a new cohort) on 15th April.
Drug Treatments	A single treatment was given in the months May to October to all lambs. No drug resistance was detected or reported. No mention of drug treatments to parental ewes.	Drug treatments are given to all lambs on the first day of each month (May to October). We assume a single day drug effect, which reduces the within host parasite stages each by 95%.<br>No treatments are given to the parental Ewes.
Weaning	Weaning occurred at 3 or 4 months of age (mid-July to mid-August).	Weaning occurred on 1st August, whereby the parental ewes (Cohort 1) is removed from the simulation.
Movement	Lambs were moved onto new pasture at weaning.	Lambs were moved onto new pasture (patch) at weaning.
Pasture contamination	The fields are described as having transmission for the prior 10 years	We implement pre-contaminated pasture (patches), detailed in the simulation R scripts.
Immunity	N/A	We assume the immunity of the lambs is reduced to account for their immaturity (the exposure-immunity relationship in the GLOWORM model was developed for adult livestock), with a reduction of 25%.<br><br>We assume Ewe immunity is reduced to account for the stresses of birth, such that immunity is reduced by 50%.

The output from the model validation against the Stear study is given in Figure S5.1. It shows good performance for the overall shape, by capturing the overarching peaks, and comparative magnitude of each peak. For example the increasing peak sizes from May to July, and a decline and partial plateauing of the peak sizes between August and September.

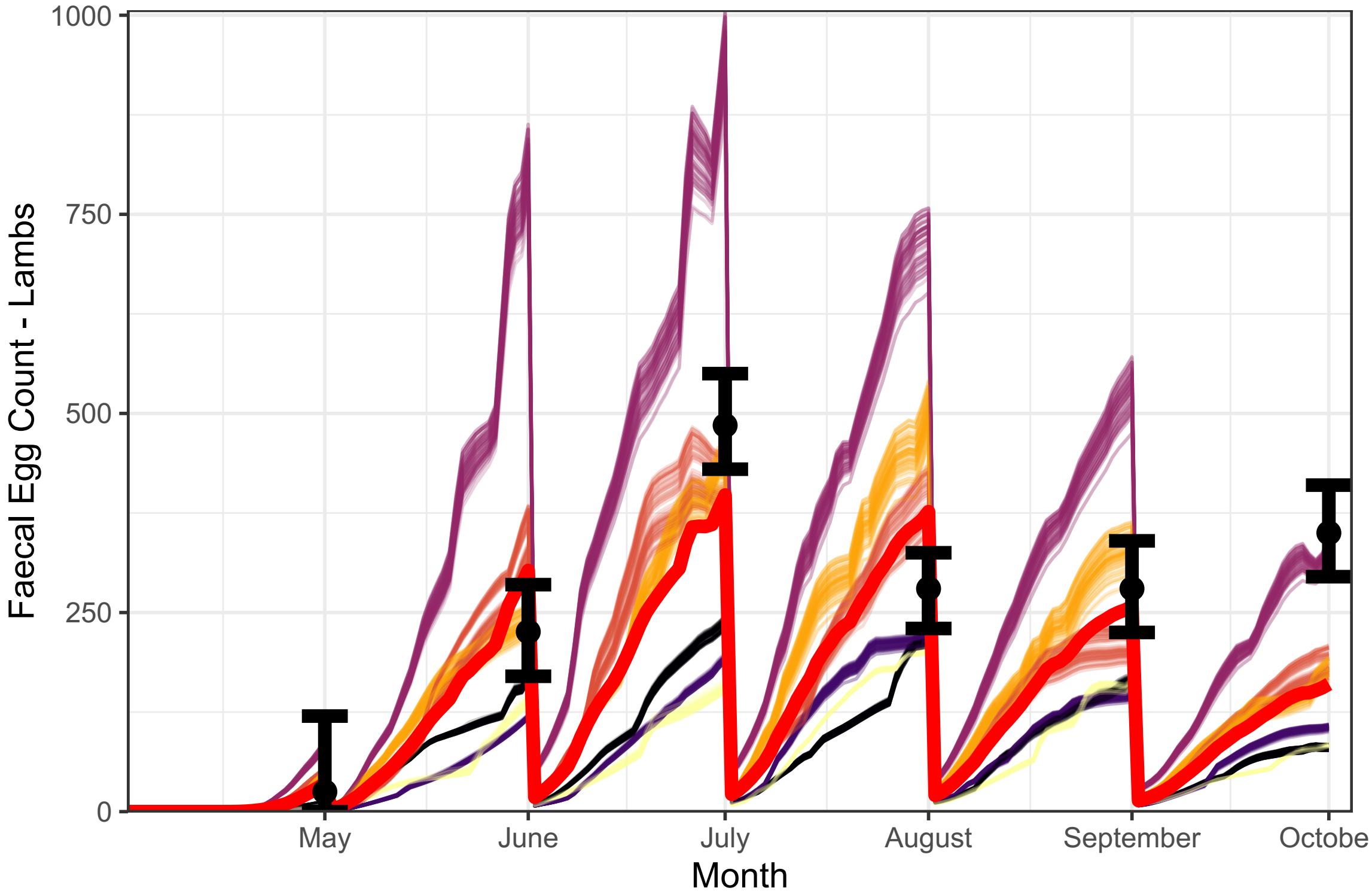

Fig. S5.1. Validation of GLOWORM Using the Stear Study. The black point and bars the mean and 95% confidence intervals from the Stear Study (Sargison et al., 2012). The thicker red line is the mean of the GLOWORM validation simulations (n = 378 simulations, 63 locations x 5 years). The thinner coloured lines are the outputs from each individual simulation, with the colour indicating year.

S5.3 Validation against Sargison Study

We next attempt to validate the GLOWORM simulation framework against the Sargison Study (Sargison et al., 2012). In brief, this study compared giving moxidectin to parental ewes as either an oral drench (~5-week residual activity) or a long-acting injectable (~16-week residual activity) to control the peri-parturient rise in FEC. With lambs then receiving three rounds of treatment with ivermectin. The oral drench (OD) and long-acting injectable (LA) groups were kept on separate fields, and we therefore conduct simulations for each group separately. The study replicated twice, so there are two OD groups and two LA groups. We simulate two cohorts, a ewe cohort and a lamb cohort. We attempt to replicate the results presented in Figure 2 in Sarginson et al., 2006. We summarise the conditions in Table S5.2 noting in particular any key time-frames or values with which we use to set up our simulations.

Table S5.2 Validation of GLOWORM using the Stear Study		
Study Aspect	Sargison Study	GLOWORM Simulations
Parasite species	*T. circumcincta*<br>*Nematodirus battus*	*T. circumcincta*
Geolocation and weather	Southeast Scotland	We assume the study was in an area bounded by the latitude and longitude coordinates of: latitude: min = 55.58, max = 55.8; and longitude min = -3.46, max = -3.12. We sample locations in the latitude and longitude direction at 0.02 intervals, giving a total of 216 sampled geolocations. Extract the weather data for these coordinates using the openmeteo R package (Pisel, 2023)
Timeframe	The year of the study was 2010. Sample collection began 18th March and ended 26th August.	We extracted weather data from the year 2010.<br>Simulations started March 1st to allow for a short burn-in to allow the FECs in the parental ewes to reach the desired level (~600 FEC). Simulations ended 26th August, therefore simulations had a total duration of 215 days.
Lambing	Lambing occurs between the 29th March and 15th April.	We take a midpoint of 7th April as the lambing day, when all lambs are born (and use this to create a new cohort).
Drug Treatments	Parental Ewes: One group received a moxidectin oral drench (~ 5-week residual activity), the other group received moxidectin as a long-acting injectable (~16-week residual activity). The treatments were given between 29th March and 15th April.<br>All lambs received ivermectin on the days 13th May, 10th June and 19th July. The FECRT for the four groups was 55%, 0%, 47%, and 0%; giving a mean across all four groups of 25.5%.	Ewes (Cohort 1):<br>For the oral drench, treatment on 7th April, which has a parasite kill efficacy of 95% for 35 days.<br>For the long-acting injectable, receive treatment on 7th April, which has a parasite kill efficacy of 95% for 35 days.<br>All lambs (Cohort 2) receive treatment on days 13th May, 10th June and 19th July. The treatment has a single day parasite killing efficacy of 0.745 (calculated as 1 minus the mean FECRT).
Weaning	Weaning occurred 109 days after April 15th.	Weaning occurred 109 days after April 15th, whereby the parental ewes (Cohort 1) are removed from the simulation.
Movement	Prior to turnout the Ewes and lambs were housed. Turnout occurred between 29th March to 15th April.	We take a midpoint of 7th April as the turnout day. From here they move from a patch where transmission does not occur to standard patch.
Pasture contamination	Pasture is assumed to be contaminated given previous needs for anthelmintic treatment of the flocks.	We implement pre-contaminated pasture (patches), detailed in the simulation R scripts.
Immunity	N/A	We assume the immunity of the lambs (Cohort 2) is reduced to account for their immaturity (the exposure-immunity relationship in the GLOWORM model was developed for adult livestock), with a reduction of 25%.<br>We assume Ewe immunity (Cohort 1) is reduced to account for the stresses of birth, such that immunity is reduced by 50%.

Figure S5.2 shows the simulated model output when compared to the Sargison Study (Sargison et al., 2012). The model performs well in capturing the overarching behaviour of the system, with the FEC rising shortly after lambing, before gradually declining. The mean values for the long-acting injectable (Figure S5.2, right panel), are marginally lower than for the Oral Drench (left panel), after the effect of the oral drench is no longer active. This difference is very slight.

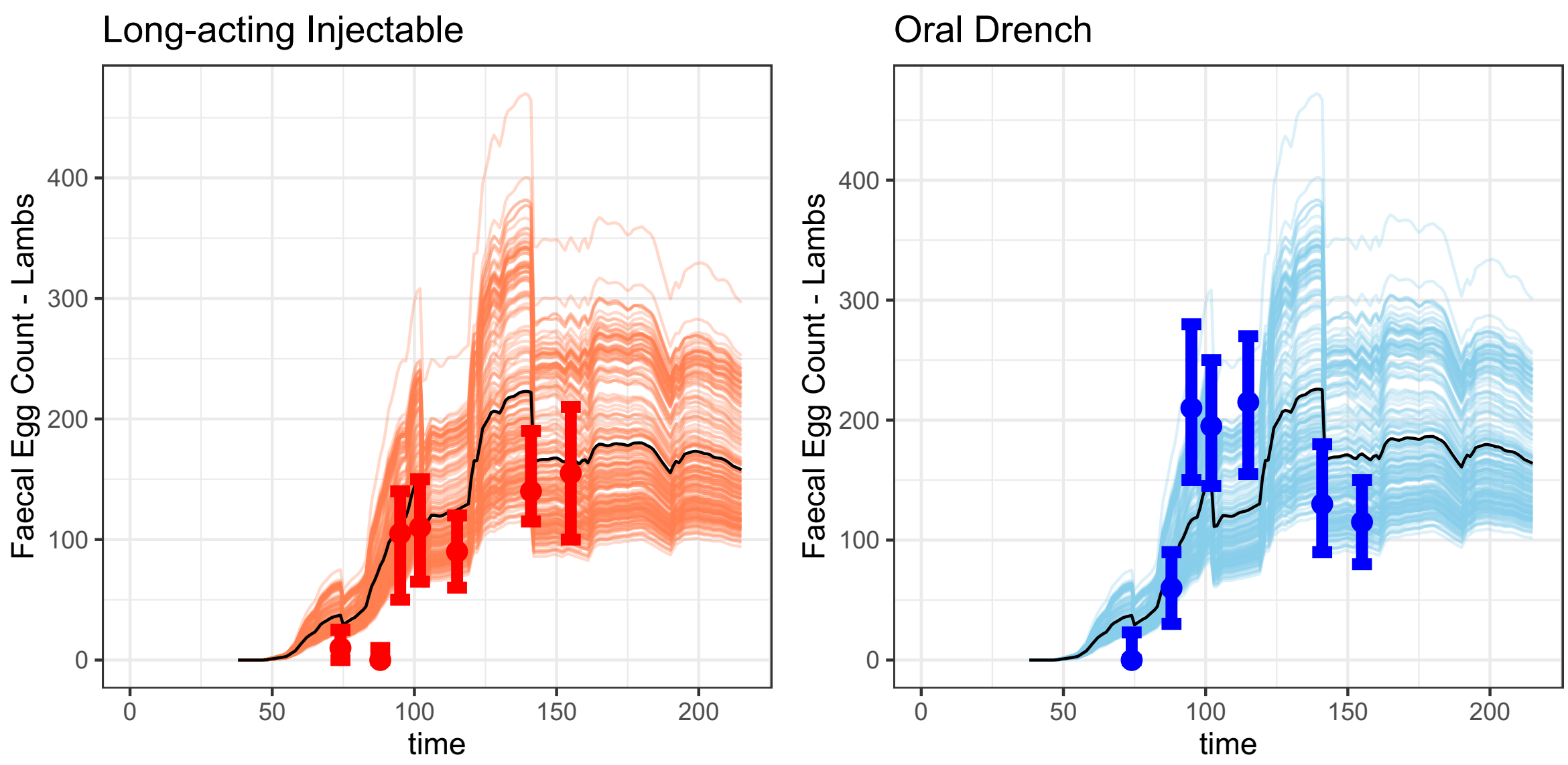

Fig. S5.2. Validation of GLOWORM Using the Sargison Study. Left Plot: This is the validation against the long-acting injectable, where the red point and bars are the mean and 95% confidence intervals from the Sargison Study (Sargison et al., 2012), and the light red lines are the output from each individual GLOWORM simulation, with the black line being the mean from those simulations (n=216). Right Plot: This is the validation against the oral drench, where the blue point and bars are the mean and 95% confidence intervals from the Sargison Study (Sargison et al., 2012), and the light blue lines are the output from each individual GLOWORM simulation, with the black line being the mean from those simulations (n=216).

S5.4 Validation against Waller Study

We next validate the GLOWORM simulation framework against the Waller Study (Waller and Thomas, 1975), for the parasite *Haemonchus contortus*.

**Table S5.4 Validation of GLOWORM using the Waller Study**		
Study Aspect	Waller Study	GLOWORM Simulations
Parasite species	*H. contortus* monitored, but mentioned other species present (worm burdens not given)	*H. contortus*
Geolocation and weather	Northumberland (Cockle Park Experimental Park)	Latitude: 55.2027 to 55.2178 (by 0.002)<br>Longitude: -1.7095 to -1.6667, (by 0.005<br><br>Extract the weather data for these coordinates using the openmeteo R package (Pisel, 2023)
Timeframe	April 1973 to November 1973	Simulations initiate on 1st April 1973 (day 1) and end on the 30th November 1973.
Lambing	16 April 1973 (estimated, as weaning occurred at approximate 14 weeks).	All lambs are born on 16th April 1973, with the creation of Cohort 2, which is the lamb cohort.
Drug Treatments	N/A	N/A
Weaning	23rd July 1973	The parental ewe population (Cohort 1) is removed on 23rd July 1973.
Movement	No details of plot movements.	Remain on the same patch throughout the simulations.
Pasture contamination	Pasture assumed to be uncontaminated as plots used had not had sheep for the prior four years.	We implemented clean pastures, where all free-living stages were absent on the first day.
Immunity	N/A	We assume the immunity of the lambs (Cohort 2) is reduced to account for their immaturity (the exposure-immunity relationship in the GLOWORM model was developed for adult livestock), with a reduction of 25%.<br><br>We assume Ewe immunity (Cohort 1) is reduced to account for the stresses of birth, such that immunity is reduced by 50%.

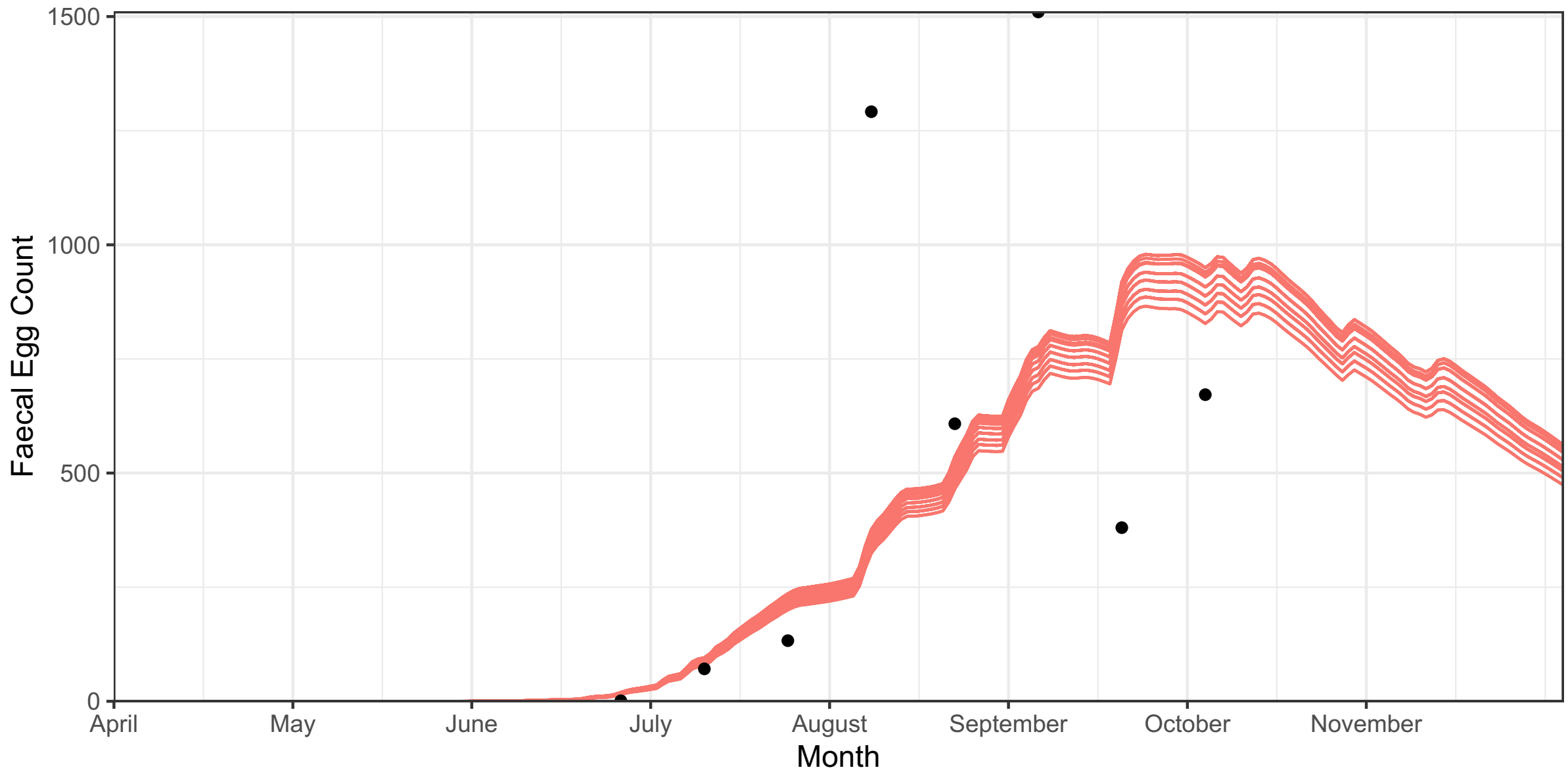

Fig. S5.3. Validation of GLOWORM Using the Waller Study. Lines are the simulated faecal egg counts of the lambs. Points are the faecal egg counts from the Waller Study (estimated using plotdigitiser).

Comments on validation:

The validation provides a good fit during the early phase of the infection. Bisects the later points; but as no confidence intervals were provided in the Waller study, given the variability of these points over a short timeframe, it could be assumed the mean values lies within these ranges and therefore the model still matches the overarching transmission dynamics of *H. contortus.*

References

Hastings, I.M., 2001. Modelling parasite drug resistance: lessons for management and control strategies. Tropical Med Int Health 6, 883–890. https://doi.org/10.1046/j.1365-3156.2001.00800.x

Helps, J.C., Paveley, N.D., White, S., Den Bosch, F., 2020. Determinants of optimal insecticide resistance management strategies. J. Theor. Biol 503, 110383.

Ingle, O.K., Melville, L.A., Beechener, S., Hardy, C., Howell, A., Hobbs, N.P., Mahon, N., Morgan, E.R., Bartley, D., Vineer, H.R., 2025. GLOWORM-META: modelling gastrointestinal nematode metapopulation dynamics to inform cattle biosecurity research. International Journal for Parasitology 55, 695–720. https://doi.org/10.1016/j.ijpara.2025.07.001

Levick, B., South, A., Hastings, I.M., 2017. A Two-Locus Model of the Evolution of Insecticide Resistance to Inform and Optimise Public Health Insecticide Deployment Strategies. PLOS Comput. Biol 13, 1005327.

Madgwick, P.G., Kanitz, R., 2022. Modelling new insecticide-treated bed nets for malaria-vector control: how to strategically manage resistance? Malar. J 21, 102.

Pisel, T., 2023. openmeteo: Retrieve Weather Data from the Open-Meteo API.

Reichert, E., Yaesoubi, R., Rönn, M.M., Gift, T.L., Salomon, J.A., Grad, Y.H., 2023. Resistance-minimising strategies for introducing a novel antibiotic for gonorrhoea treatment: a mathematical modelling study. The Lancet Microbe 4, e781–e789. https://doi.org/10.1016/S2666-5247(23)00145-3

REX Consortium, 2013. Heterogeneity of selection and the evolution of resistance. Trends Ecol. Evol 28, 110–118.

Rose, H., Wang, T., van Dijk, J., Morgan, E.R., 2015. GLOWORM-FL: A simulation model of the effects of climate and climate change on the free-living stages of gastro-intestinal nematode parasites of ruminants. Ecological Modelling 297, 232–245. https://doi.org/10.1016/j.ecolmodel.2014.11.033

Rose Vineer, H., Howell, A., Smith, Z., Tollett, A., Stubbings, L.A., Glover, M.J., Hobbs, N., Archer, D., Graham-Brown, J., Gilleard, J., 2025. Sustainable control of parasites in ewes (SCOPE): pre-registration of protocol for a randomised controlled trial on the use of moxidectin in ewes at lambing time. https://doi.org/10.31220/agriRxiv.2025.00307

Rose Vineer, H., Verschave, S.H., Claerebout, E., Vercruysse, J., Shaw, D.J., Charlier, J., Morgan, E.R., 2020. GLOWORM-PARA: a flexible framework to simulate the population dynamics of the parasitic phase of gastrointestinal nematodes infecting grazing livestock. International Journal for Parasitology 50, 133–144. https://doi.org/10.1016/j.ijpara.2019.11.005

Sargison, N.D., Bartram, D.J., Wilson, D.J., 2012. Use of a long acting injectable formulation of moxidectin to control the periparturient rise in faecal Teladorsagia circumcincta egg output of ewes. Veterinary Parasitology 189, 274–283. https://doi.org/10.1016/j.vetpar.2012.04.020

Stear, M.J., Abuagob, O., Benothman, M., Bishop, S.C., Innocent, G., Kerr, A., Mitchell, S., 2006. Variation among faecal egg counts following natural nematode infection in Scottish Blackface lambs. Parasitology 132, 275–280. https://doi.org/10.1017/S0031182005009029

Waller, P.J., Thomas, R.J., 1975. Field studies on inhibition of *Haemonchus contortus* in sheep. Parasitology 71, 285–291. https://doi.org/10.1017/S0031182000046722
